# Evolution of Gene Regulatory Networks by Fluctuating Selection and Intrinsic Constraints

**DOI:** 10.1371/journal.pcbi.1000873

**Published:** 2010-08-05

**Authors:** Masaki E. Tsuda, Masakado Kawata

**Affiliations:** Division of Ecology and Evolutionary Biology, Graduate School of Life Sciences, Tohoku University, Sendai, Japan; MRC Laboratory of Molecular Biology, University of Cambridge, United Kingdom

## Abstract

Various characteristics of complex gene regulatory networks (GRNs) have been discovered during the last decade, e.g., redundancy, exponential indegree distributions, scale-free outdegree distributions, mutational robustness, and evolvability. Although progress has been made in this field, it is not well understood whether these characteristics are the direct products of selection or those of other evolutionary forces such as mutational biases and biophysical constraints. To elucidate the causal factors that promoted the evolution of complex GRNs, we examined the effect of fluctuating environmental selection and some intrinsic constraining factors on GRN evolution by using an individual-based model. We found that the evolution of complex GRNs is remarkably promoted by fixation of beneficial gene duplications under unpredictably fluctuating environmental conditions and that some internal factors inherent in organisms, such as mutational bias, gene expression costs, and constraints on expression dynamics, are also important for the evolution of GRNs. The results indicate that various biological properties observed in GRNs could evolve as a result of not only adaptation to unpredictable environmental changes but also non-adaptive processes owing to the properties of the organisms themselves. Our study emphasizes that evolutionary models considering such intrinsic constraining factors should be used as null models to analyze the effect of selection on GRN evolution.

## Author Summary

Various organismal traits, including the morphology of multicellular species and metabolism in unicellular species, are determined by the amount and combinations of proteins in the cell. The complex regulatory network plays an important role in controlling the protein profiles in a cell. Recent studies have revealed that gene regulatory networks have many interesting structural and mutational features such as their scale-free structure, mutational robustness, and evolvability. However, why and how these features have emerged from evolution is unknown. In this paper, we constructed an evolutionary model of gene regulatory networks and simulated its evolution under various environmental conditions. The results show that most features of known gene regulatory networks evolve as a result of adaptation to unpredictable environmental fluctuations. In addition, some internal organismal factors, such as mutational bias, gene expression costs, and constraints on expression dynamics, are also important for GRN evolution observed in real organisms. Thus, these GRN features appear to evolve as a result of not only adaptation to unpredictable environmental changes but also non-adaptive processes owing to the properties of the organisms themselves.

## Introduction

The genetic basis of organismal evolution is one of the fundamental problems in biology [1]–[7]. The modes of selection for phenotypes would influence the fixation probabilities of the mutations that affect the phenotypes [8], and the profile of the mutations fixed during the course of evolution would determine the architecture of the genomes and the genetic systems underlying the phenotypes [9]. However, because genetic systems would modify the phenotypic effects of the mutations, the properties of the genetic system would influence the rates and directions of phenotypic evolution as well as the mutational robustness and evolvability [10]–[15]. Therefore, both phenotypes and genetic systems have evolved by mutually influencing each other.

Gene regulatory networks (GRNs) constitute important parts of such genetic systems and are involved in various biological processes such as environmental responses in unicellular organisms and cell differentiation in multicellular organisms [4], [16], [17]. Recent theoretical and experimental studies have revealed that complex GRNs have evolved by successive gene duplication, changes in regulatory interactions, and particularly in prokaryotes, horizontal gene transfer [18]–[20]. In addition, recent studies have addressed the structural features of complex GRNs such as redundancy, scale-free outdegree distributions and exponential indegree distributions [4], [21]–[24] and the contribution of these features to genetic characteristics such as mutational robustness and evolvability [25]–[29].

One important question with regard to the evolution of complex GRNs is the evolutionary origin of these structural and mutational properties. Various evolutionary processes simultaneously influence GRN evolution and these properties are interrelated. It is thus difficult to identify the factors that have promoted the evolution of these properties, which could evolve as a result of being directly influenced by selection and also incidentally as a result of other factors [30]–[33]. Thus, to identify the factors responsible for the evolution of the properties of complex GRNs, it is necessary to consider not only selection but also various mutational processes and constraining processes.

Selection for phenotype is one of the most important driving forces of organismal evolution. However, the impact of phenotypic selection on the evolution of GRNs is unclear. The mode of selection strongly influences the fate of mutations and the profile of mutations fixed during the course of evolution ultimately determines the architecture of GRNs. Thus, it is important to examine how different modes of phenotypic selection would affect the evolution of GRNs. However, there are significant limitations to our general understanding of the processes of adaptation in evolutionary biology. Many previous studies on the evolution of mutational robustness with respect to GRNs have focused on the fixation of phenotypically neutral mutations under stabilizing selection with a constant optimal environment [25], [34]. On the other hand, the fixation of beneficial mutations for phenotypic adaptation under changing environments is limited [29].

Many studies have suggested that some examples of GRN architectures are related to mutational robustness and evolvability [11], [26], [35], [36]. Theoretical studies have proposed that these genetic properties appear to be evolvable traits [29], [37]–[40] and that these genetic properties could play a significant role in organismal evolution [41]. However, it is unclear how mutational robustness and evolvability influences the process of GRN evolution.

Certain properties of GRN might have evolved through non-adaptive processes such as mutations and biophysical constraints on gene regulation [30], [31], [42]–[45]. Mutations in particular is the ultimate source of genetic variation. Thus, the biased properties of mutations can potentially influence the tendency of an organism to evolve. For example, the probability of a transcription factor binding site formation as a result of mutations could vary by several orders of magnitude mainly owing to the extensive variation in the size of potential *cis*-regulatory regions among organisms [31], [46], and the rate of gene deletion could be several times higher than the rate of gene duplication in certain organisms [47], [48]. Moreover, it has been suggested that the horizontal transfer of regulatory genes is observed to a lesser extent than that of phenotypic genes [20]. Several studies have suggested that certain characteristic features of complex GRNs, such as redundancy and scale-free degree distributions could evolve as an inevitable outcome of mutations [30], [31]. However, these previous studies have not considered certain essential evolutionary processes such as selection and gene duplication. It is therefore unclear whether such characteristic features of complex GRNs evolved as a result of selection or as a result of the inherent properties of the mutations.

The purpose of this study was to identify the evolutionary causes of various structural and mutational properties of complex GRNs, such as redundancy, indegree and outdegree distributions, mutational robustness, and evolvability. For this purpose, we constructed an individual-based model of GRN that dynamically controls gene expression levels and allows populations to evolve under various fluctuating conditions of selection with various kinds of mutations such as gene duplication and deletion, *cis*-, *trans*-regulatory mutation and horizontal gene transfer. In this study, to explore selective conditions that promote the evolution of complex GRNs, we first examine the evolution of GRNs under various conditions of fluctuating selection. Second, for showing the adaptive mechanisms for the evolution of complex GRNs, we examine the fitness effect of all the mutations that arose during the evolution. Third, to explore whether internal factors of organisms promote or inhibit the evolution of GRNs, we examined the impact of gene expression cost, constraints on expression dynamics, and several types of mutational biases such as the relative rates of gene duplication and deletion, the possibility of formation of new transcription factor binding sites and horizontal gene transfers. Finally, on the basis of the results of the above analyses, we discuss the major evolutionary causes of various properties of complex GRNs, i.e., redundancy, scale-free out-degree distributions, exponential in-degree distributions, mutational robustness, and evolvability.

## Results

### Outline of the model

**Figure 1:**
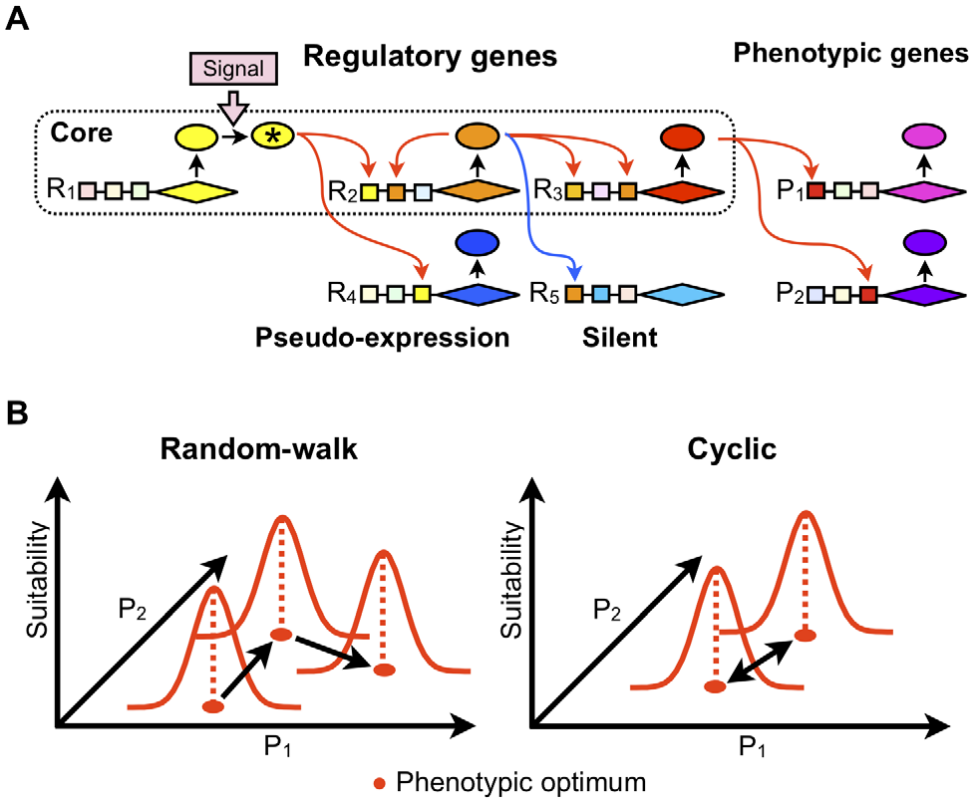
Schematic representation of the model. (A) Each gene has a *cis*-regulatory region composed of 100 *cis*-sites (boxes; potential transcription factor binding sites) and a coding region (diamonds) from which products (circles) of the genes are created. The products of regulatory genes would bind to the corresponding binding sites (represented by the same colors) and control the expression of the target genes. A *cis*-regulatory region is allowed to have multiple binding sites for the same transcription factor; thus, the strength of regulatory interactions, including activation (red arrows) and repression (blue arrows), depend on the numbers and properties of the binding sites. The regulatory cascade would start by imposing an input signal that activates the *R*_1_ gene. The phenotype of an individual is defined as the steady-state expression level of phenotypic genes. Core genes are expressed and actually involved in phenotypic expression. On the other hand, pseudo-expression genes are expressed but not involved in phenotypic expression. (B) The fitness of an individual depends on the cost of gene expression and the phenotypic suitability to the environment. The phenotypic suitability to the environment depends on the Euclidian distance between the individual phenotype and the optimum phenotype. The position of the optimum shifts a constant distance away (*d*) at every certain generation (*f^−1^*) in a random direction (random-walk) or to a fixed position (cyclic).

Before presenting the results, we provide a brief description of our model (see Methods for details). The model represents a single regulatory module that controls gene expression in response to specific external stimuli (Fig. 1A). We assume that the populations comprise haploid asexually reproducing individuals. Individuals have their own genomes, and a genome of an individual determines the individual's GRN structure. Individuals of a population at generation = 0 are clonal and have 10 regulatory genes (*R*_1_, … , *R*_10_) and 2 phenotypic genes (*P*_1_, *P*_2_) and the GRN of an individual has a random structure. The expression levels of each gene are restricted to a range of [0.0, 10.0]. The phenotype of an individual is defined as the combination of steady-state expression levels of phenotypic genes. Thus, an individual phenotype is represented as a vector, 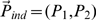.

Individuals reproduce according to their fitness value. A fitness value of an individual depends on phenotypic selection and the cost of gene expression. The phenotypic selection is defined as a Gaussian function, where an individual phenotype that is closer to an arbitrarily defined optimum has higher fitness (Fig. 1B). When an offspring is produced, various types of mutations such as gene duplication, gene deletion, *cis*- and *trans*-regulatory mutation, basal transcription level mutation, and horizontal gene transfer are expected to occur with certain probabilities. Under given simulation conditions, a population is allowed to evolve for 50,000 generations, and 60–100 replicated populations are examined under a simulation conditions. Throughout the simulation studies, a set of parameter values is used as a standard set of conditions (Table 1). Then, in order to examine the influence of a certain factor on GRN evolution, only 1 parameter value is changed while the other parameters are kept at standard values. The standard values are determined by approximating those of yeast because of the availability of appropriate yeast data [49], [50].

**Table 1:** Glossary of parameters and their standard values.

Parameter	Description	Standard val.
*Z*	Population size	10^5^
*M_ini_*	Number of phenotypic genes in the founder individual	2
*N_ini_*	Number of regulatory genes in the founder individual	10
*L*	Number of cis-sites in a cis-regulatory region	10^2^
*m*	Size of DNA sequences that are recognized by a transcription factor.	7.14
*n*	Possible number of DNA motifs that are produced by *m* base pairs of DNA sequence	9950
*c*	Fitness load per unit of gene expression	10^−5^
*V*	Level of steady-state constraints on the phenotypic expression	10^−4^
*μ_BTL_*	Basal transcription-level mutation rate (per gene per generation)	10^−6^
*μ_CIS_*	*Cis*-regulatory mutation rate	10^−6^
*μ_TRA_*	*Trans*-regulatory mutation rate	10^−6^
*μ_DEL_*	Gene deletion rate	10^−6^
*μ_DUP_*	Gene duplication rate	10^−6^
*μ_HOR_*	Horizontal gene transfer rate	0

### The evolution of GRNs under fluctuating selection

To elucidate the selective conditions for the evolution of complex GRNs, we first examined the evolution of GRN under various conditions of fluctuating phenotypic selection. For that purpose, we compared the structures of GRNs after simulation runs for 50,000 generations with standard parameter values under various fluctuating conditions of phenotypic selection. The fluctuation of phenotypic selection was modelled by shifting the position of an optimal phenotype by generation. The initial position of the optimum was set as the phenotype of founding individuals at generation = 0. We assumed 2 types of optimum shift, a random-walk and a cyclic optimum shift for exploring the impact of the difference in the direction of the optimum shift (Fig. 1B). For both types of optimum shift, we analyzed the optimum shifts with various amplitudes (*d*) and frequencies (*f*). In the random-walk optimum shift, the optimum shifts away from the previous position by a constant distance (*d*) in a random direction for each 1/*f* generation. In the cyclic optimum shift, there are 2 alternative optima that are spaced at a constant distance (*d*), and the optimum is switched from one to another for each 1/*f* generation.

**Figure 2:**
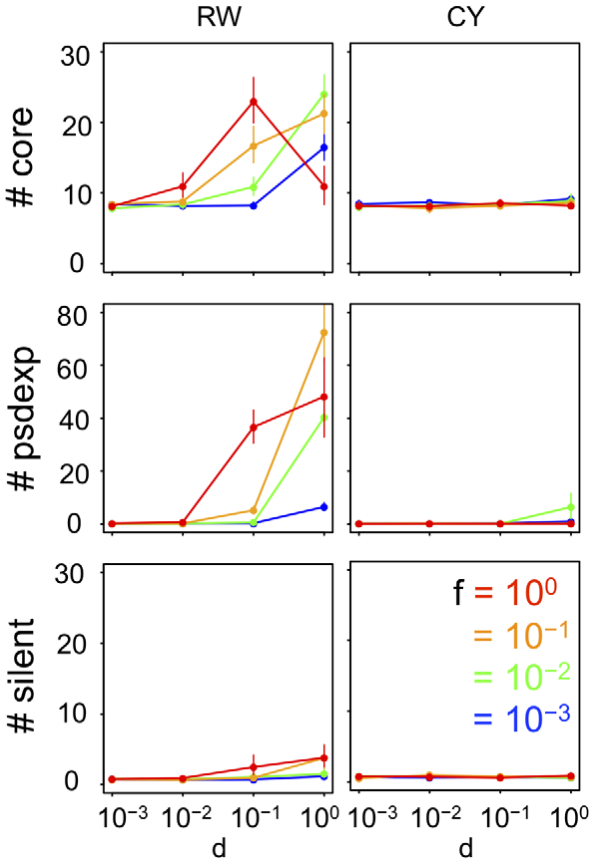
GRN structures that evolved under various fluctuations of phenotypic selection. The number of core genes (#core), pseudo-expression genes (#psdexp), and silent genes (#silent) in GRNs that evolved for 50,000 generations under random-walk optimum shift (RW), and those that evolved under cyclic optimum shift (CY). All parameters were set at standard values (Table 1). Each point connected by solid lines represents the mean number of each type of genes in evolved GRNs under each selective condition. Vertical bars attached to the point represent 95% confidence intervals. *d* and *f* represent the amplitude and frequency of the optimum shift, respectively.

Figure 2 shows the structures of GRNs after 50,000 generations of evolution under various fluctuations of phenotypic selection. As a proxy for the GRN structure, we first examined the number of regulatory genes that were responsible for the expression of phenotypic genes (denoted as core genes in Fig. 1A). In our model, not all regulatory genes were responsible for the expression of phenotypic genes because some regulatory genes were not transcribed. An example of such an untranscribed gene is the *R5* gene in Fig. 1A (silent). In other cases, regulatory genes did not regulate phenotypic genes either directly or indirectly. An example of this is the *R4* gene in Fig. 1A (pseudo-expression).

The results show that under the random-walk optimum shift, the number of core and pseudo-expression genes in evolved GRNs increases with the increase in amplitudes (*d*) and frequencies (*f*) of the optimum shifts. However, under the cyclic optimum shift, GRNs with a slightly large number of regulatory genes evolve only when the optimum shift has high amplitude and low frequency. While the random-walk optimum shift with higher amplitude and frequency tends to promote the evolution of complex GRNs, the number of both core genes in the evolved GRN is relatively small when both the amplitude and the frequency are extremely high.

**Figure 3:**
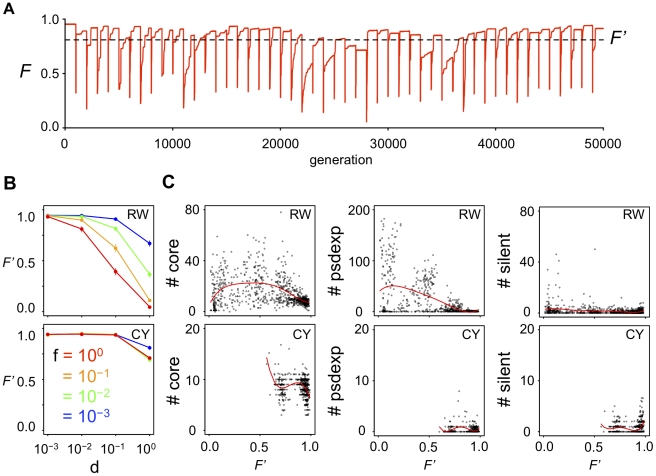
Relationship between the time-averaged fitness of a population and the GRN structures. (A) An example of the changes of the mean fitness in a population during evolution. Red line indicates the mean fitness of a population at certain generation. Horizontal dotted line indicates the time-averaged fitness (*F′*) during the evolution in this population. (B) The time-averaged fitness of GRNs that evolved under various fluctuations of phenotypic selection. (C) The relationship between the time-averaged fitness and the structure of GRNs. Red line indicates the fitting curve to the quintic equation by non-linear least square method.

To clarify the relationship between the intensity of optimum fluctuation and the evolution of complex GRNs, we analyzed the time-averaged fitness from the 0 generation to the 50,000^th^ generation in each population (Fig. 3A). Because the time-averaged fitness of a population becomes smaller as the intensity of optimum fluctuation becomes stronger, the time-averaged fitness of a population may be used as a good indicator of the intensity of optimum fluctuation (Fig. 3B).

We examined the relationship between the time-averaged fitness during evolution and the structure of GRNs after evolution (Fig. 3C). The results show that the maximum number of core genes is observed when the time-averaged fitness is at a middle level. The results indicate that the evolution of complex GRNs is most efficiently promoted when the intensity of the optimum fluctuation is moderate. However, the evolution of complex GRNs is disturbed when the intensity of optimum fluctuation becomes too strong.

Generally, populations with low fitness would be exposed to a high risk of extinction in nature. Thus, realistically, complex GRNs would evolve under a moderately strong optimum shift, e.g., small and frequent (d = 10^−1^, f = 10^−1^) optimum shift in a random direction or a large and infrequent (d = 10^0^, f = 10^−3^) optimum shift in both random and cyclic directions. On the other hand, simple GRNs would evolve under a small and infrequent optimum shift (d = 10^−3^, f = 10^−3^), and this selective condition corresponds to a pure stabilizing selection with a fixed optimum.

Thereafter, to examine the relationships between GRN structures and mutational properties such as the mutational robustness and evolvability, we examined the phenotypic effect of various types of mutations after simulation runs for 50,000 generations. For that purpose, a single mutation was introduced into an individual and then the phenotypes of mutant individuals were compared with those of the original individuals. One thousand randomly chosen individuals in a population were examined for each type of mutation. In addition, to clarify the multilateral aspects of mutational robustness and evolvability, we classified the mutations into 3 types according to their phenotypic effect. The *Non-effect* mutations cause no phenotypic changes. The *Loss-of-phenotype* mutations cause loss of the expression level at least one phenotypic gene (*P_i_*<10^−2^) or prevent the expression from reaching a steady state. *Significant* mutations cause phenotypic changes but do not also produce the effect of a *Loss-of-phenotype* mutation. In addition, we measured the size of phenotypic changes caused by *Significant* mutations. Only the results of mutations against core genes are presented here since mutations against non-core genes generally have no phenotypic effect (data not shown).

**Figure 4:**
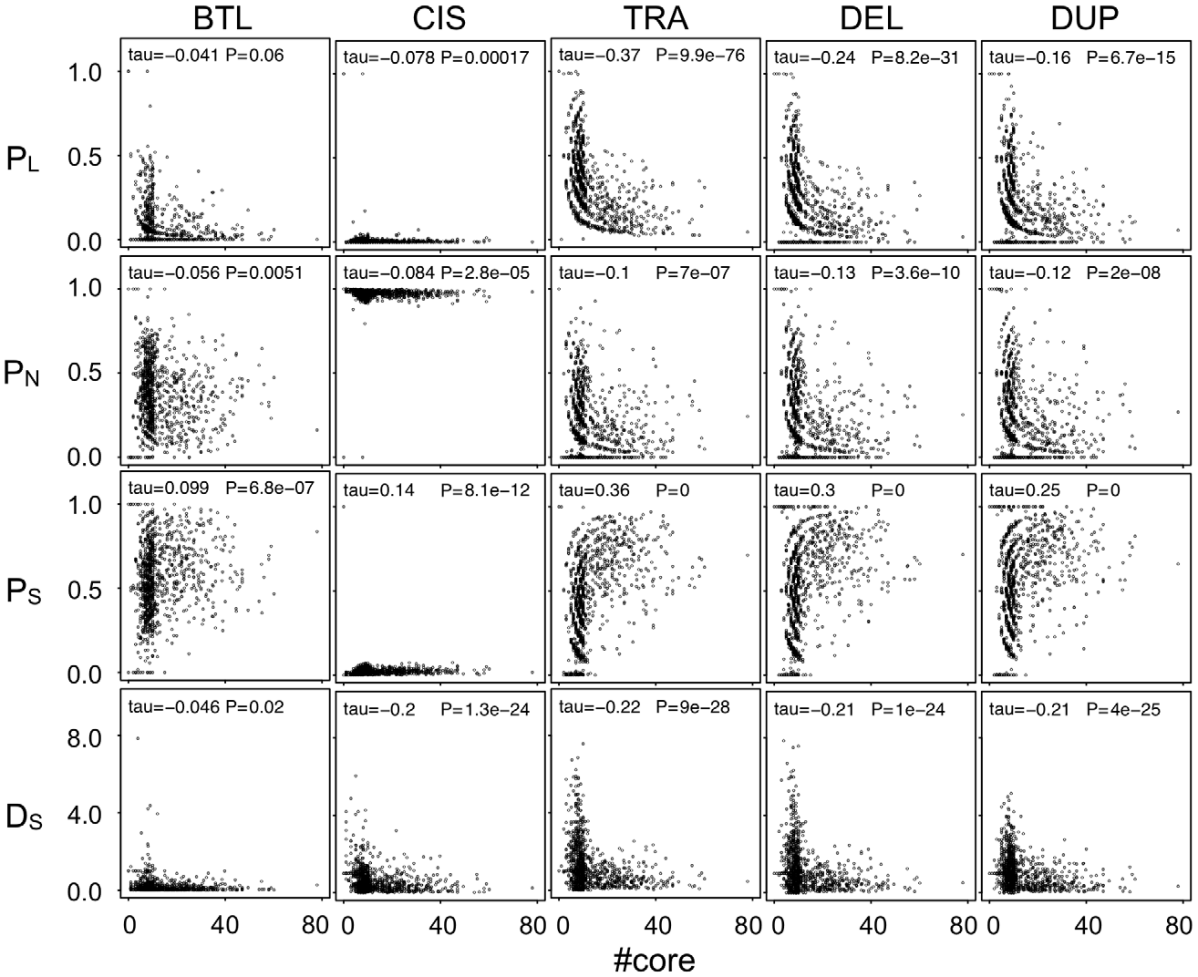
Relationship between the number of core genes in GRNs and the phenotypic effects of various types of mutations in core genes. Points represent the results of each population evolved under various amplitudes (*d*) and frequencies (*f*) of random-walk optimum shift. Horizontal axes indicate the number of core genes in a population. Panels in each column indicate the effect of different types of mutations (basal transcription level mutation, BTL; *cis*-regulatory mutation, CIS; trans-regulatory mutation, TRA; gene deletion, DEL; gene duplication, DUP). *P_L_*, *P_N_*, and *P_S_* show the proportion of mutations that cause *Loss-of-phenotype*, those that have no phenotypic change (*Non-effect*), and those that have a *Significant* phenotypic change, respectively (*P_L_* + *P_N_* + *P_S_* = 1). *D_S_* shows the size of phenotypic changes caused by *Significant* mutations (the Euclidean distance between the original and mutant phenotypes). Statistical significance of the correlation was analyzed by Kendall's correlation test.

Figure 4 shows the relationships between GRN structures and the phenotypic effect of mutations in evolved GRNs under various conditions of phenotypic selection. Several tendencies were derived from the results. First, *trans*-regulatory mutations, gene deletion, and gene duplication have similar effects, and these mutations are unlikely to represent *Loss-of-phenotype* mutations in complex GRNs. Second, most of the *cis*-regulatory mutations were *Non-effect* mutations, while most of the other types of mutations were rarely *Non-effect* mutations. Third, the extent of phenotypic changes caused by *Significant* mutations was generally small in complex GRNs.

These results suggest that the complex GRNs confer both mutational robustness, i.e., a low proportion of *Loss-of-phenotype* mutations (*P_L_*) and small phenotypic changes in *Significant* mutations (*D_S_*)) as well as evolvability, i.e., high proportions of *Significant* mutations (*P_S_*) and a high mutational target size. On the contrary, simple GRNs have low evolvability. i.e., low *P_S_* and small mutational target size and fragility, i.e., high *P_L_* and large *D_S_*. However, because the mutational target size is small in simple GRNs, spontaneous mutations are less likely to arise. Thus, although simple GRNs are fragile when a mutation is artificially introduced such as a gene knockout in the laboratory, they are robust to spontaneous mutations under natural conditions.

Then, to confirm whether mutational target sizes and *P_S_* are associated with actual evolvability, we examined the rates of phenotypic adaptation in response to a benchmark selective condition by using populations obtained after 50,000 generations of evolution (see Methods). The results show that both the mutational target sizes and *P_S_* are positively correlated with the rates of phenotypic adaptation (Fig. S1). Thus, the mutational target size and *P_S_* examined in laboratory experiments could be a good indicator of actual evolvability.

From the present results, we suggest that the evolvability of a target phenotype in a population could be defined as follows:
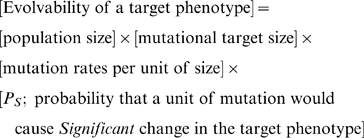
(1)where a unit of mutation is a gene in this study. Because mutations occurring in core genes were considered in this analysis, a mutational target size in this analysis is the number of core genes, and the *P_S_* value is the probability of *Significant* mutations occurring with respect to core gene mutations.

### Adaptive mechanism for the evolution of complex GRNs

In this section, we analyze adaptive mechanisms explaining the present results where a certain mode of fluctuating selection remarkably promotes the evolution of complex GRNs. In the present study, complex GRNs have high evolvability, which is positively correlated with mutational target sizes and *P_S_*. Because high evolvability is considered to be favorable under conditions of fluctuating selection [29], [39], it is possible that the evolution of complex GRNs is promoted by its high evolvability. Thus, we first analyzed the evolution of GRNs without the influence of evolvability. To remove the influence of evolvability, we controlled the mutational target size and *P_S_*. The mutations in the present model were assumed to occur at a per-gene mutation rate, so that individuals with large numbers of genes (i.e., large mutational target size) had high mutation rates per individual. Thus, we kept per-individual mutation rates constant regardless of the number of regulatory genes (see Method for details). The results showed that the effect of constant mutation rates per individual is almost the same as the assumption of constant mutation rates per gene (Fig. S2). Then, to remove the influence of *P_S_*, we set *P_S_* at 1 regardless of the difference in GRN structures (*P_S_* = 1, *P_L_* = *P_N_* = 0; see Methods for detail). The results were almost the same as those obtained without controlling the *P_S_* value (Fig. S3). These results indicate that evolution of complex GRNs in our model could be promoted without the influence of evolvability and that the influence of evolvability on GRN evolution might be small.

**Figure 5:**
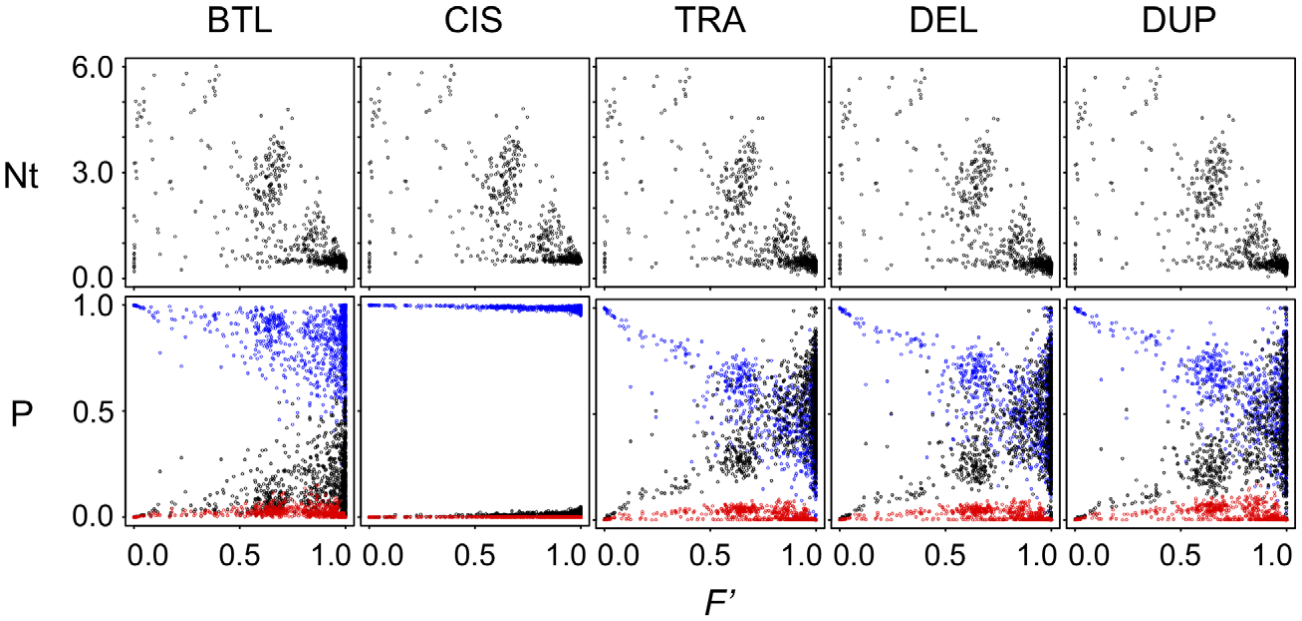
Relationship between the intensity of the optimum fluctuation and the fitness effect of various types of mutations during evolution. Points represent the results of a population that evolved under various conditions of random-walk optimum shift. Horizontal axes indicate the time-averaged fitness of a population. Panels in each column indicate the effect of different types of mutations (same as Figure 4). *Nt* indicates the total number of mutations that arose during the evolution for each types of mutations. *P* indicates the proportions of mutations that have beneficial (red), neutral (blue), and deleterious (black) effects, respectively.

The other possible mechanism for complex GRNs is fixations of beneficial mutations through phenotypic adaptation. To elucidate how the mutations contribute to the phenotypic adaptation, we analyzed fitness effects (i.e., a difference in fitness between a mutant and its original individual) for all the mutations that arose during the 50,000 generations of evolution in each population. In this analysis, we removed the cost of gene expression (c = 0) from the original model since we wanted to obtain the fitness effects caused only by the differences in phenotypes. Figure 5 shows the relationships between the intensity of optimum fluctuation and the fitness effects of various kinds of mutations. The results showed that mutations are likely to be beneficial when the fluctuation is at a moderate level (Fig. 5 red points). On the contrary, mutations are likely to be neutral when the fluctuation is strong (Fig. 5 blue points) and are likely to be deleterious when the fluctuation is weak (Fig. 5 black points). The results indicate that the evolution of complex GRNs is caused by the fixation of beneficial mutations through phenotypic adaptation.

**Figure 6:**
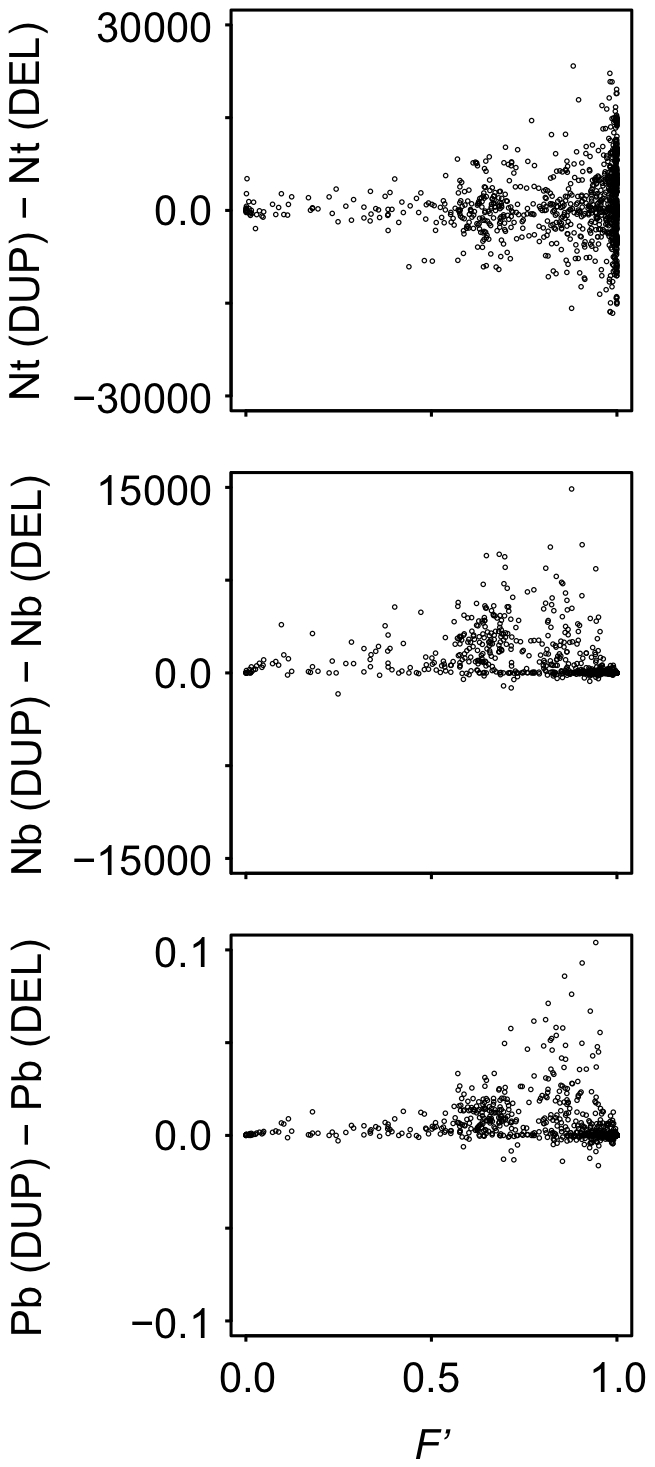
Relationship between the intensity of the optimum fluctuation and the fitness effects of gene duplication and gene deletion during evolution. Points represent the results of a population that evolved under various conditions of random-walk optimum shift. *Nt*(*x*), *Nb*(*x*) and *Pb*(*x*) indicate the total number of mutations, number of beneficial mutations, and the proportions of beneficial mutations that arose during the evolution for mutation type *x*, respectively. Vertical axes indicate the difference in the number and the proportions of beneficial mutations between gene duplications and gene deletions. Horizontal axes indicate the time-averaged fitness of a population (*F′*).

**Figure 7:**
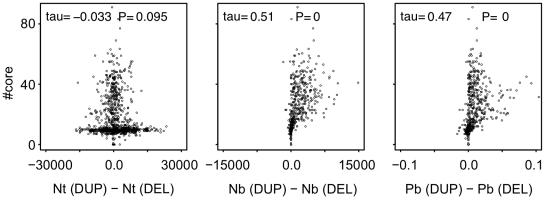
Relationship between the number of core genes after evolution and the number of beneficial gene duplications and gene deletions. Points represent the results of a population that evolved under various conditions of random-walk optimum shift. Vertical axes indicate the number of core genes. *Nt*(*x*), *Nb*(*x*) and *Pb*(*x*) indicate the total number of mutations, the number of beneficial mutations, and the proportions of beneficial mutations that arose during the evolution for mutation type *x*, respectively. Horizontal axes indicate the difference in the number and the proportions of beneficial mutations between gene duplications and gene deletions. Statistical significance of the correlation was analyzed by the Kendall's correlation test.

Then, we examined the difference in the number of gene duplications and deletions that showed beneficial fitness effects, because fixation rate of gene duplications need to be greater than those of gene deletion for the evolution of complex GRNs. The results show that gene duplications are more likely to be beneficial than gene deletions particularly when the optimum fluctuation is moderate (Fig. 6). We then examined the relationship between the number of core genes in the evolved GRN and the number of beneficial gene duplications and gene deletions that occurred during the evolution. The results show that the number of core genes in GRN becomes larger as the number of beneficial gene duplications become more than those of gene deletions (Fig. 7). These results indicate that the evolution of complex GRNs are promoted mainly by phenotypic adaptations acquired through the more frequent fixation of beneficial gene duplication than through gene deletion.

### Influence of constraining factors

The above analysis showed that the fixation of beneficial gene duplication by phenotypic selection is an important adaptive factor for promoting evolution of complex GRNs. However, the genes included in the complex GRNs of the above analysis are generally too abundant to be regarded as a single regulatory module. Thus, it is reasonable to expect the existence of certain constraining factors to restrict the evolution of complex GRNs in real organisms. We examined the impact of certain examples of internal constraining factors that are inherent in organisms, such as (i) the functional constraints on gene expression dynamics, (ii) cost of gene expression, and (iii) the biased properties of mutations, in the subsequent analysis.

#### (i) Functional constraint on expression dynamics

**Figure 8:**
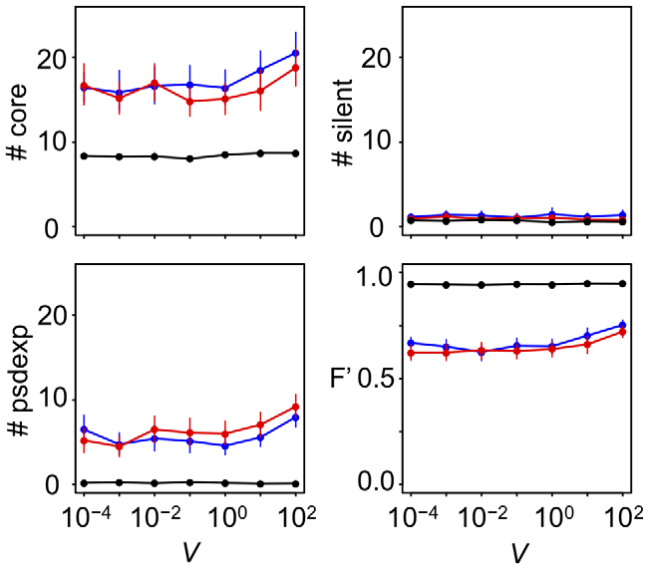
Effect of the strength of steady-state constraints on GRN evolution. Greater values of *V* indicate weaker constraints on steady-state expression (*V* = 10^−4^, standard parameter value). Points connected by solid lines represent the mean number of core genes (#core), pseudo-expression genes (#psdexp), silent genes (#silent) and the time-averaged fitness (*F′*) in populations that evolved for 50,000 generations under each simulation condition. Vertical bars indicate 95% confidence intervals. Different colors indicate different conditions of phenotypic selection: d = 10^−1^, f = 10^−1^ (red); d = 10^0^, f = 10^−3^ (blue); d = 10^−3^, f = 10^−3^ (black) under random-walk optimum shift.

The present model assumes that offspring are not viable if expression of phenotypic genes does not reach a steady state. This assumption could constrain the process of GRN evolution. The mode and degree of constrains on the expression dynamics of a regulatory module might depend on a functional context that the regulatory module is involved in. For example, a regulatory module that work in early developmental stage might be under strong demand for steady-state expression, on the other hand, those that work in later developmental stage might be under relatively weak demand for steady-state expression. To examine whether steady-state constraints on gene expression affect the evolution of a complex GRN, we conducted simulations with steady-state constraints (*V*) of varying strength. The results indicate that strong steady-state constraints slightly restrict the evolution of a complex GRN under conditions of fluctuating selection (Fig. 8). The results indicate that functional constraints on the expression dynamics of GRNs could affect the evolution of GRNs.

#### (ii) Cost of gene expression

**Figure 9:**
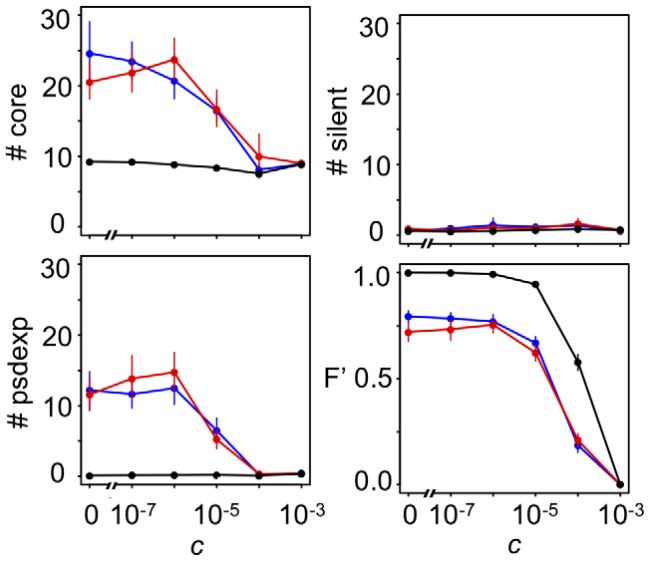
Effect of gene expression costs on GRN evolution. Greater values of *c* indicate the larger fitness load of a unit of gene expression (*c* = 10^−5^, standard parameter value). Points connected by solid lines represent the mean number of core genes (#core), pseudo-expression genes (#psdexp), silent genes (#silent) and the time-averaged fitness (*F′*) in populations that evolved for 50,000 generations under each simulation condition. Vertical bars indicate 95% confidence intervals. Different colors indicate different conditions of phenotypic selection: d = 10^−1^, f = 10^−1^ (red); d = 10^0^, f = 10^−3^ (blue); d = 10^−3^, f = 10^−3^ (black) under random-walk optimum shift.

It has been suggested that the cost of gene expression has a significant impact on the evolution of gene expression [51]–[53]. Thus, here, to examine how strongly the cost of gene expression would affect the evolution of GRNs, we conducted simulations with various fitness loads per unit of gene expression (Fig. 9). The result showed that larger cost of gene expression significantly prohibited the evolution of complex GRNs, but the level of cost completely prohibiting the evolution of complex GRNs was seemed to be unrealistic, because no population could be sustained under such extremely high level cost even if the optimum fluctuation was very weak. These results suggest that the fluctuations in phenotypic selection could promote the evolution of complex GRN when the fitness load of gene expression cost is realistic level.

#### (iii) Mutational bias

We next examined the influence of mutational bias on GRN evolution. In other words, we wanted to determine the properties of GRNs that are more sensitive to mutational bias. Lynch (2007) proposed that scale-free degree distributions evolved as a result of mutational bias in the relative rate of gain and loss of regulatory interactions under pure stabilizing selection [31]. However, this study did not consider mutations causing changes in the GRN size, i.e., gene duplication and deletion. Both type of mutations that change regulatory interactions and those that change the size of the GRN play a central role for the evolution of degree distributions [19]. Thus, in the present work we examined 3 types of mutational processes; (a) the relative rate of gain and loss of regulatory interactions; (b) the relative rate of gene deletion and duplication; and (c) horizontal transfer of regulatory genes.

#### (iii-a) Relative rate of gain and loss of regulatory interactions

To examine the mutational bias with respect to the gain and loss of regulatory interactions, we used a derived parameter *C_mut_* that is defined as the probability that a binding site of a specific transcription factor is present in a *cis*-regulatory region (see Methods for detail). For controlling the value of *C_mut_*, the size of a *cis*-regulatory region (*L*) was varied. A greater value of *L* indicates a higher probability of binding sites formation through regulatory mutations (*C_mut_*). According to the estimations of Lynch (2007) [31], we roughly inferred the order of *C_mut_* as 10^−3^–10^−2^ for prokaryotes, 10^−2^–10^−1^ for unicellular eukaryotes, and 10^−1^–10^0^ for multicellular eukaryotes. In addition, each GRN in our model, which represents a single regulatory module, is too small to obtain smooth degree distributions. Thus, to measure the degree distributions, we created an assembled GRN for each simulation conditions by considering the regulatory modules of 100 replicate populations of each simulation condition as a single global GRN (i.e., a single assembled GRN is composed of 100 separated regulatory modules).

**Figure 10:**
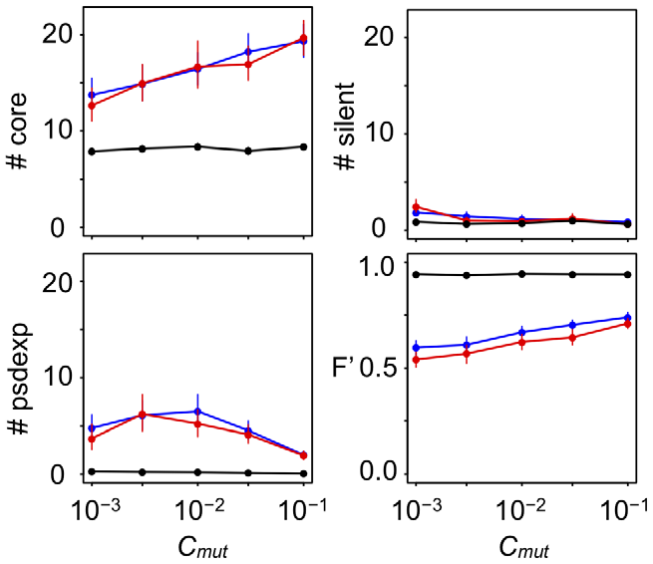
Effects of the probability of binding site formation by regulatory mutations (*C_mut_*) on GRN evolution. Greater values of *C_mut_* indicate larger probabilities of binding site formation by regulatory mutation (*C_mut_* = 10^−2^, standard parameter values). To control the *C_mut_* value, the size of the *cis*-regulatory region of a gene (*L*) was varied; *L* = 10, 30, 100, 303, and 1000 for *C_mut_* = 10^−3^, 3×10^−3^, 10^−2^, 3×10^−2^, and 10^−1^, respectively. Points connected by solid lines represent the mean number of core genes (#core), pseudo-expression genes (#psdexp), silent genes (#silent) and the time-averaged fitness (*F′*) in populations that evolved for 50,000 generations under each simulation condition. Vertical bars indicate 95% confidence intervals. Different colors indicate different conditions of phenotypic selection; d = 10^−1^, f = 10^−1^ (red); d = 10^0^, f = 10^−3^ (blue); d = 10^−3^, f = 10^−3^ (black) under random-walk optimum shift.

Figures 10–12 show the number of core genes and the in- and outdegree distributions with various values of *C_mut_*. The results show that the number of core genes and the shape of the indegree distributions in complex GRNs are affected by the changes in the values of *C_mut_*, while the outdegree distributions were mostly unaffected by the changes. A smaller value of *C_mut_* tends to decrease the number of core genes. However, fluctuating phenotypic selection could promote the evolution of complex GRNs even under such small values of *C_mut_* (Fig. 10).

**Figure 11 pcbi-1000873-g011:**
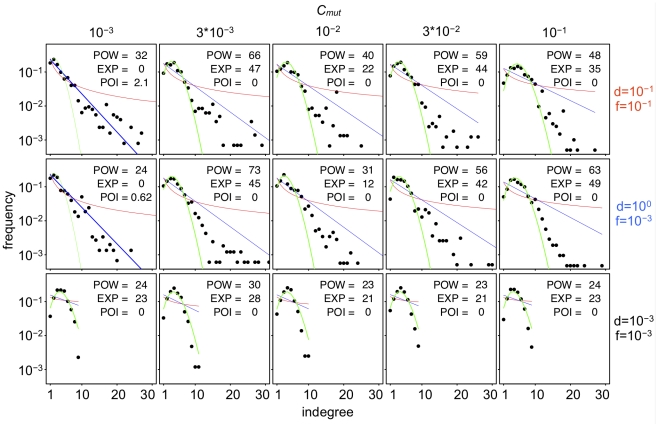
Indegree distribution of the assembled GRNs that evolved under various *C_mut_* levels. Horizontal and vertical axes in each panel show the indegree (the number of regulatory interactions that arrived at a gene) and the frequency, respectively. Note that the vertical axes are shown logarithmically to demonstrate the exponential character of the distribution. Different rows and columns indicated the different conditions of phenotypic selection and different values of *C_mut_*, respectively. Lines in each panel indicate the regression of the plot to the Power law distribution (red), exponential distribution (blue), and Poisson distribution (green). Regression was estimated by a nonlinear least-square method. To judge the goodness of regression, Akaike's information criterion (AIC) was used, and the regression that showed the smallest value of AIC was drawn as a thick line. POW, EXP and POI in each panel indicate the differences between AIC value of the best regression model and those of power-law (scale-free), exponential and poisson distributions, respectively.

**Figure 12:**
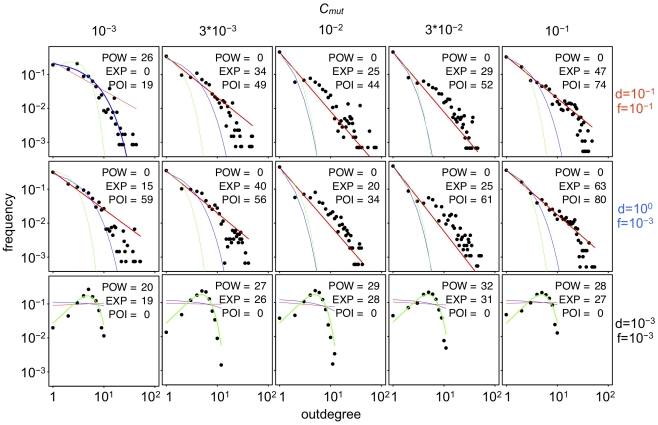
Outdegree distribution of assembled GRNs that evolved under various *C_mut_* levels. Horizontal and vertical axes in each panel show the outdegree (the number of regulatory interactions that depart from a gene) and the frequency, respectively. Note that the both horizontal and vertical axes are shown logarithmically to demonstrate the scale-free character of the distribution. Different rows and columns show the different conditions of phenotypic selection and the different values of *C_mut_*, respectively. Lines in each panel indicate the regression of the plot to the Power law distribution (red), exponential distribution (blue) and Poisson distribution (green). Regression was estimated by a nonlinear least-square method. To judge the goodness of the regression, Akaike's information criterion (AIC) was used, and the regression that showed the smallest value of AIC was drawn as a thick line. POW, EXP and POI in each panel indicate the differences between AIC value of the best regression model and those of power-law (scale-free), exponential and poisson distributions, respectively.

The shape of indegree distributions generally well fit to the Poisson distribution rather than exponential distribution; however, some complex GRNs that evolved under low *C_mut_* levels had exponential indegree distributions, as observed in real microorganisms (Fig. 11). On the other hand, indegree distributions that evolved under high *C_mut_* levels had the Poisson distribution with a single peak (Fig. 11).

Contrary to the indegree distributions, the shapes of the outdegree distributions were only correlated with the number of core genes rather than *C_mut_*, where GRNs with a larger number of core genes had scale-free outdegree distributions as observed in real microorganisms, while simple GRNs did not (Fig. 12). These results suggest that the scale-free outdegree distribution is a product of complex GRNs that evolve by phenotypic selection rather than because of the influence of mutation properties. On the other hand, the exponential indegree distributions are caused not only by phenotypic selection but also by the mutation properties.

#### (iii-b) Relative rate of gene duplication and deletion

**Figure 13:**
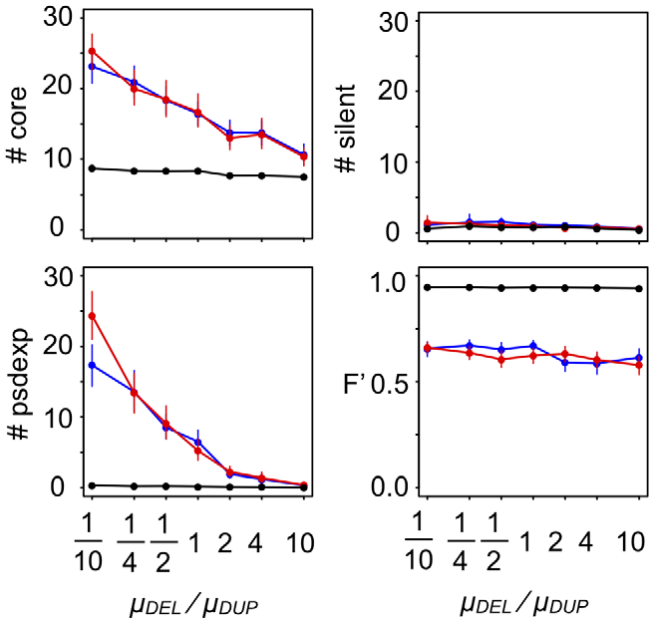
Relationships between GRN structures and the relative rates of gene duplication and gene deletion (*μ_DEL_/μ_DUP_*). Standard parameter value, *μ_DEL_/μ_DUP_* = 1. To control the value of (*μ_DEL_/μ_DUP_*), only *μ_del_* are varied from 10^−7^ to 10^−5^, while *μ_dup_* was fixed at a standard value (10^−6^). Points connected by solid lines represent the mean number of core genes (#core), pseudo-expression genes (#psdexp), silent genes (#silent) and the time-averaged fitness (*F′*) in populations that evolved for 50,000 generations under each simulation condition. Vertical bars indicate 95% confidence intervals. Different colors indicate different conditions of phenotypic selection; d = 10^−1^, f = 10^−1^ (red); d = 10^0^, f = 10^−3^ (blue); d = 10^−3^, f = 10^−3^ (black) under random-walk optimum shift.

Several studies have proposed that gene deletion rates could be several times higher than gene duplication rates [47], [48]. Thus, the mutation bias might disturb the evolution of a complex GRN. Figure 13 shows the effect of mutational bias on the relative rate of gene deletion and gene duplication on the number of regulatory genes of the GRN. The result shows that as the gene deletion rates increase with respect to gene duplication rates, the number of core genes in evolved GRNs tends to decrease. However, even when deletion rates are an order of magnitude higher than duplication rates, fluctuating phenotypic selection could lead to the evolution of complex GRNs (Fig. 13). Inversely, even when duplication rates are an order of magnitude higher than deletion rates, phenotypic selection with weak optimum fluctuation effectively prohibited the evolution of complex GRNs.

#### (iii-c) Horizontal gene transfer

**Figure 14:**
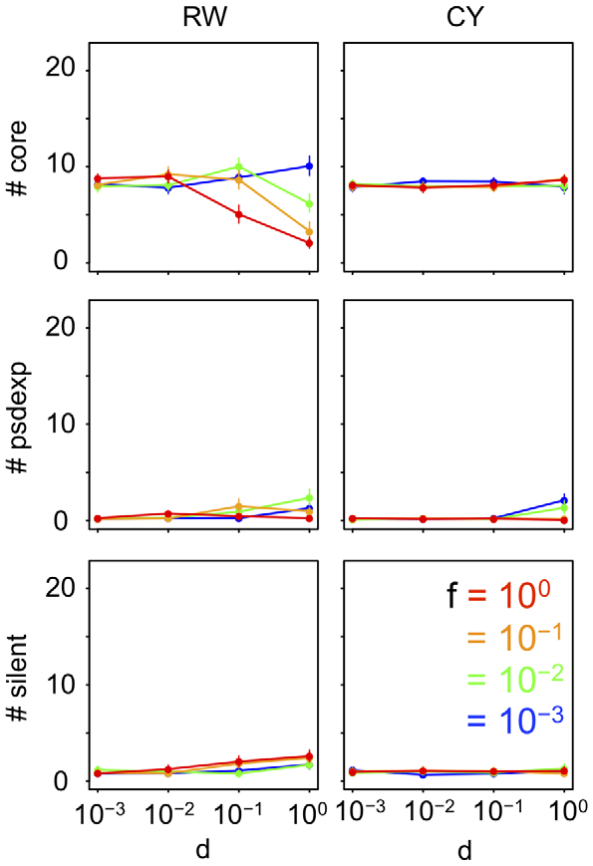
GRN structures that evolved with horizontal transfer of regulatory genes. Instead of the duplication of existing regulatory genes, a randomly created new regulatory gene was introduced into a GRN (i.e., *μ_DUP_* = 0, *μ_HOR_* = 10^−6^). All other parameters were set at standard values. Each point connected by solid lines represents the mean number of each type of genes in evolved GRNs under each selective condition. Vertical bars attached to the point represent 95% confidence intervals. *d* and *f* represent the amplitude and frequency of the optimum shift, respectively.

Horizontal transfers of regulatory genes are observed less frequently than those of phenotypic genes [20], however, it is unclear that whether the phenomenon was owing to the properties of selection or mutation. Thus here, to examine the effect of horizontal gene transfer on GRN evolution, a randomly created new regulatory gene was added to a GRN instead of duplicating an existing gene (i.e., *μ_HOR_* = 10^−6^, *μ_DUP_* = 0). The results showed that horizontal transfer of regulatory genes was not maintained in GRNs under any conditions of phenotypic selection (Fig. 14). This result indicates that maintenance of horizontal transfer of regulatory genes is much more difficult than those of duplications, and the absence of horizontally transferred regulatory genes is explained by the inherent properties of the mutation rather than by the differences in phenotypic selection.

## Discussion

### Adaptive mechanism for the evolution of GRNs under fluctuating phenotypic selection

Our study showed that the mode of fluctuation in phenotypic selection has a remarkable impact on the evolution of GRNs. In particular, it was found that fluctuating phenotypic selection with random-walk optimum shift strongly promotes the evolution of complex GRNs with high mutational robustness and evolvability. On the other hand, phenotypic selection with cyclic optimum shift contributes only slightly under limited conditions. By examining a fitness effect of all the mutations during evolution, our study has determined that phenotypic adaptation by beneficial gene duplication represents a major factor that promotes the evolution of complex GRNs.

Our study has shown that evolution of complex GRNs is promoted when the intensity of optimum fluctuation is moderate. This phenomenon was thought to occur because the fitness effects of *Significant* mutations depends upon the intensity of optimum fluctuation (see Fig. S10 for illustration). When the fluctuation is weak, most phenotypic changes produced by mutations are likely to be deleterious because the phenotype of the current population is very close to optimal and mutated phenotypes are more likely to be far from optimal. Thus, phenotypic selection tends to inhibit the fixation of the mutations under these conditions. When the intensity of the optimal fluctuation is moderate, certain phenotypic changes produced by mutations would be beneficial since the mutated phenotypes have a greater chance of being located closer to the optimum than the current population. Thus, phenotypic selection tends to promote the fixation of the mutations under these conditions. When the fluctuation is strong, the position of the optimum would be too far from both the current population and the mutated phenotypes. Thus, most phenotypic changes induced by the mutations would not change the fitness. Such phenotypic changes are selectively neutral and would be fixed only by genetic drift. Thus, phenotypic selection does not play any role in the fixation of mutations under these conditions.

We observed the relationship between phenotypic effects (*Significant*, *Non-effect*, and *Loss-of-phenotype*) and the fitness effects (beneficial, neutral, and deleterious) of mutations. *Non-effect* mutations are always neutral by definition. *Loss-of-phenotype* mutations are usually deleterious because the movement of the optimum was assumed to avoid the vicinity of 0.0 in our model. On the contrary, *Significant* mutations could show all three of the fitness effects. Particularly, *Significant* mutations that cause only small phenotypic changes are not exactly neutral, but mutations having very small fitness effects are known to behave like neutral mutations. Such mutations are referred to as “nearly neutral”. Thus, our study regards the mutations as neutral when their fitness effects are smaller than 10^−2^. The exact judgment on near neutrality theoretically depends upon the population size and the selection differential. In our analyses, we did not adopt a precise judgment with respect to neutrality since it was difficult to calculate selection differential precisely. We defined values ranging from 10^−4^ to 10^−1^ as indicating neutrality, and the results were qualitatively unaffected by changing these values (data not shown).

We analyzed the fitness effect of a mutation at the time of incidence of the mutation in a population. However, because the position of the optimum fluctuates over generations, the fitness effect estimated in our analysis was not a complete indicator for judging the fate of a mutation, particularly when the fluctuation was very frequent. Although such a factor might make it more difficult to detect the fitness effects of the mutation, the present analysis showed high statistical significance. Thus, we believe that the analysis is valid and that the mechanism explained above would operate for most populations of our simulation. Although the detailed mechanisms of how gene duplication is more likely to become beneficial than gene deletions under conditions of fluctuating selection are unclear, we can conclude that there should be differences in the phenotypic effects between gene duplication and deletion. To address this problem, we need to perform detailed analyses with regard to the sizes and directions of phenotypic changes caused by mutations.

### Role of genetic drift in GRN evolution

Fixation of a mutation occurs not only through selection but also through genetic drift. A role of genetic drift in the fixation of a mutation becomes stronger when the efficiency of selection becomes weak. The fixation probabilities of mutations by genetic drift depend on the mutation rates, i.e., mutations that occur more frequently will be fixed more frequently than the other kinds of mutations. Thus, for the evolution of complex GRNs through genetic drift, the following two conditions must be satisfied: (i) gene duplications occur more frequently than gene deletions; (ii) selection is ineffective for fixation (i.e., very small population size, weak strength of selection, and strong optimum fluctuation). This study showed that when the intensity of optimum fluctuation was weak, evolution of complex GRNs was effectively restricted even when gene duplications occur more frequently than gene deletions (Fig. 13). This indicates that the effects of selection were much larger than those of genetic drift, and thus, the conditions where the evolution of complex GRNs is promoted only by genetic drift might be limited.

### Beneficial effect of loss-of-function by *trans*-regulatory mutations

The complex GRNs not only had a larger number of core genes but also had pseudo-expression genes. In our study, pseudo-expression genes are produced by loss-of-function because of *trans*-regulatory mutations. These results indicate that phenotypic changes occurring through loss-of-function by *trans*-regulatory mutations are likely to contribute to phenotypic adaptation. Loss-of-function mutations are generally considered deleterious in molecular evolution. However, our study showed that a loss-of-function mutation could become somewhat beneficial when one of the duplicated genes loses its function under conditions of fluctuating selection.

### Pseudo-expression genes and silent genes as a genomic architecture

Our study showed that the evolution of GRNs under various selective and constraining conditions would produce not only core genes but also non-core genes. While silent pseudogenes have been commonly observed in various species, only a small number of silent regulatory genes were observed in our model. This might be because the loss of gene expression by basal transcription level mutations and *cis*-regulatory mutations rarely occurred in our model. Silencing of gene expressions through loss-of-function mutations at the transcription factor binding sites and the promoter regions were commonly observed in real organisms. As far as we know, while the actual rates of these mutations were unknown, the mutations might occur more frequently than those in our simulations.

Moreover, most non-core genes in our model were pseudo-expression genes. This might be because these pseudo-expression genes were produced by loss-of-function through *trans*-regulatory mutations, and the mutations were likely to become beneficial under fluctuating selection in our model. Although such pseudo-expression genes might be wasteful, recent studies have revealed that significant fractions of non-coding RNA are composed of transcribed pseudo-genes [54], [55]. Thus, the presence of pseudo-expression genes in GRNs in this study is not necessary unrealistic, and the transcribed pseudo-genes present in real organisms might be products of adaptation of gene expression under fluctuating selection.

While pseudo-expression genes and silent genes do not involved in functional parts of GRNs, these competent constitute significant part of genomic contents. Thus, our study indicates that the mode of phenotypic selection could influence not only GRN structures but also genomic architectures.

### Importance of dosage effect of mutations in the evolution of gene expression

While *cis*-regulatory mutations have been receiving considerable attention in the studies on gene expression evolution, various other mutations, including gene duplications, gene deletions, and *trans*-regulatory mutations could also influence gene expression through dosage effects. Although dosage effects of gene duplication and deletion have been well recognized, the selective conditions that promote the fixation of these mutations are unknown. Our study demonstrates that these mutations were fixed by selection when the direction of selection was randomly fluctuated. Functional protein dosage increases with gene duplications, but decreases with gene deletions and loss-of-function by *trans*-regulatory mutations; thus, these mutations appeared to become beneficial more often under fluctuating selection in this model.

While our study emphasizes the beneficial aspects of dosage effect, several studies in some multicellular organisms suggested that dosage effects negatively influence fitness. For example, small-scale duplications in some kinds of genes, such as developmental genes and transcription factors, might be limited because a quantitative balance between different proteins through molecular interactions is important for the functioning of these types of genes [56], [57]. In our analysis, the fixations of mutations that have dosage effects were strongly inhibited even if the emergence of mutations was positively biased when the intensity of optimum fluctuation was small (Fig. 13). Although our study focused on the selection by external environments, functional constraints through molecular interactions between proteins in a cell seem to be important in real organisms. Thus, it is necessary to consider such biophysical processes in future studies on GRN evolution.

### Regulatory module for specific cellular function

While, many studies about the evolution of development and GRN have focused on relatively discrete spatial and temporal changes of gene expression (i.e., heterotopy and heterochrony) where importance of *cis*-regulatory mutation is proposed to play major role [1], [3], [29], [58], [59]. Our study focused on a single regulatory module producing the continuous changes of gene expression level in a specific cellular type (heterometry [60]). Such continuous differences in the levels of gene expressions in a specific cellular type are also often correlated with the variations in fitness and quantitative traits in multicellular organisms [61]–[63]. Likewise, a steady-state gene expression level in response to specific environmental stimuli is also correlated with fitness in unicellular organisms [51], [64]–[66]. In real organisms only a part of GRN is used in a specific condition [67], and such a condition-specific sub-network appears to be a regulatory module that controls specific cellular function [17], [68], [69]. Thus, a significant part of phenotypic evolution could be represented as the quantitative changes in gene expression by single regulatory module.

### Mode of environmental fluctuation

Changes in the direction of selection for phenotypes owing to spatio-temporal environmental fluctuation is one of the major driving forces of organismal evolution [64]–[67]; however, most studies in the field of evolutionary biology are focused on evolution under stabilizing selection with fixed optimum or directional selection in a fixed direction [37], [65], [70]. Only a few studies have dealt with adaptation toward a moving optimum [8] or a cyclically fluctuating optimum [29], [71]. Interestingly, while it is difficult to elucidate historical patterns of fluctuation in the direction of selection, a study on the long-term evolutionary patterns of various quantitative traits from fossil records showed that the most of the traits fit to the evolutionary models of random-walk or stasis rather than prolonged directional selection [72]. Thus, the modes of fluctuation in phenotypic selection assumed in this paper might be plausible.

### Constraints on expression dynamics of GRNs

Our results demonstrated that steady-state constraints on GRN expression dynamics could significantly restrict the evolution of complex GRNs. This is because the constraints would decrease the proportion of mutations that could contribute to phenotypic adaptation. A previous study demonstrated that signaling pathways that evolved under constraints for different response dynamics would show the different levels of complexity [73]. This indicates that the strength of constraints might depend on the type of expression dynamics, and the result of that study might be compatible with our results.

### Gene expression cost

Our results demonstrated that the fitness load of gene expression costs significantly restricted the evolution of complex GRNs. This is because the cost of gene expression would increase both the deleterious effects of gene duplications and the beneficial effects of gene deletion. Previous studies have suggested that even small costs of gene expression have significant impacts on the evolution of gene expression in microorganisms [51]–[53]. However, the fitness loads of a single gene duplication/deletion might be generally very small; thus, the impacts of expression costs on GRN evolution have not been sufficiently studied. By using individual-based simulations that could deal with very large population sizes, we could demonstrate that even small costs of gene expression could have significant impacts on GRN evolution.

### The probability of binding site formation by mutation and the shape of degree distributions

Our study showed that some mutational bias had a considerable impact on GRN evolution. The probability of transcription factor binding site formation by regulatory mutations (*C_mut_*) mainly affected the number of core genes in GRNs and the shape of indegree distributions in complex GRNs, but not the outdegree distributions (Figs. 10–12). In particular, some GRNs that evolved with lower *C_mut_* showed exponential distributions as observed in microorganisms, while those with higher *C_mut_* showed single-peaked Poisson distributions (Fig. 11). These results indicated that the exponential indegree distributions observed in real microorganisms might be due to their small *C_mut_* rather than being a direct product of selection; in addition, indegree distributions of global GRNs in multicellular organisms might be single peaked, although only a part of the GRNs were identified in multicellular organisms. In contrast, the scale-free outdegree distribution depended on GRN complexity (i.e., phenotypic selection) but not on *C_mut_* (Fig. 12). Thus, scale-free outdegree distributions can be considered as a by-product of complex GRNs that evolved through phenotypic selection. Most studies have focused only on the scale-free feature of biological networks and have ignored the differences between outdegree and indegree distributions. Lynch (2007) [31] argued that scale-free degree distributions could evolve as a result of mutational bias where the gain rates of regulatory interactions were much smaller than loss rates (i.e., low *C_mut_*). However, the study presented only an indegree distribution rather than outdegree distributions, and the shape of the indegree distribution appeared to be exponential. Hence, our results might correspond to those of Lynch (2007).

However, our results might contain some biases since we obtained the degree distributions by assembling separated regulatory modules. If the removal of regulatory interactions between regulatory modules in real GRNs disrupts the scale-free and exponential properties of degree distributions, GRN models that contain multiple regulatory modules would be necessary. On the other hand, if the removal of regulatory interactions between regulatory modules does not disrupt the degree distributions in real GRNs, real GRNs might be regarded as the assembly of complex regulatory modules, even if there are some connections between modules.

Although the precise mechanisms for the evolution of these degree distributions were unclear from our study, gene duplications and changes in regulatory interactions by *trans*-regulatory mutations might be necessary factors for the evolution of scale-free properties. We conducted an additional analysis by changing the rates of *cis*- and *trans*-regulatory mutations. The results showed that the decreased rates of *cis*-regulatory mutation did not affect the number of core genes, indegree distributions, and outdegree distributions (Fig. S4, S5, S6). On the other hand, decreased rates of *trans*-regulatory mutations decreased the number of core genes (Fig. S4) and disrupted the shape of outdegree distributions (Fig. S6). These results imply that the gains of regulatory interactions through *trans*-regulatory mutations might contribute to the increase of core genes and the establishment of scale-free outdegree distributions.

In our results, indegree distributions mostly fitted to the Poisson distributions rather than the exponential distribution, and the distribution peaked as *C_mut_* increased. In contrast, outdegree distributions mostly fitted to scale-free distributions and the shape did not depend on *C_mut_*. We hypothesized the mechanisms of these result as follows.

The mechanism of indegree distributions: Theoretically, a random network where a link (input and output regulatory interactions) between two randomly selected nodes (genes) exists at constant probability (*C_mut_*) is supposed to have the Poisson distribution for both its indegree and outdegree distributions where the average (generally denoted as *λ*) is equal to the average degree of nodes (i.e., *λ* = *N*×C*_mut_*, where *N* is the number of nodes in the network). Because an increase of *C_mut_* would increase the average degree of nodes (*N*×C*_mut_*), the change in the shape of the indegree distributions owing to the changes in *C_mut_* seemed natural from this equation. The Poisson distribution with very low average values (*λ*<1) is almost indistinguishable from the exponential distribution, and the estimated value of *C_mut_* for organisms in which exponential indegree distribution was reported are generally small. Thus, the reported indegree distributions in these organisms might fit the Poisson distribution rather than the exponential distribution.

The mechanism of outdegree distributions: Bhan et al (2002) showed that the joint effects of node duplication and link rewiring would change the degree distributions from Poisson to scale-free [19]. While the study did not distinguish indegree and outdegree distributions (i.e. the degree was sum of the indegree and outdegree), we presume that both would be scale-free if the distributions were analyzed separately. In addition, while we assumed that the establishment of regulatory interactions depended on the *cis*-regulatory and coding regions, Bhan's study did not have such assumption. Thus, the differences we observed between indegree and outdegree distributions might be attributed to our assumption. This assumption might disrupt the changes of indegree distributions from Poisson to scale-free even if gene duplication and regulatory mutations occurred. On the other hand, we supposed that the change in outdegree distributions from Poisson to scale-free is due to the joint effects of gene duplication and *trans*-regulatory mutations in our model, because the change in outdegree distributions from Poisson to scale-free was also disrupted when the rates of *trans*-regulatory mutations became low (Fig. S6). To detect the actual mechanisms for degree distribution, more detailed examinations on how various mutations change the regulatory interactions are necessary.

### Relative rate of gene duplication and deletion

Recent studies in yeasts have revealed higher rates of gene duplication and deletion than previously thought [49] and the abundance of copy number variations in some model organisms [74]. In addition, the contributions of copy number variations to gene expression variations have also been elucidated [75]. Surprisingly, the estimation of the expected number of gene duplication and deletion per genome is even higher than those of base substitutions [49]. Furthermore, while relative rates of gene duplication and deletion were said to be biased toward a high deletion rate, the estimated duplication rate is several times higher than the deletion rate [49]. Thus, genetic drift might have a larger effect to promote the complex GRNs in real organisms.

### Horizontal gene transfers

In contrast to the relative rate of gene deletion and gene duplication, horizontal transfer of single regulatory genes did not contribute to the evolution of complex GRNs under any conditions of phenotypic selection (Fig. 14). The results indicate that duplication of regulatory genes is indispensable for the evolution of complex GRNs and also that the minority of horizontally transferred regulatory genes against phenotypic gene in bacterial species were not due to natural selection but due to an inherent property of the mutation. However, we only considered the horizontal transfer of a single regulatory gene in this model. Some studies have reported that functionally related genes are often clustered and that transcription factors and their target genes tend to exist close to each other in a genome [42], [45], [76]. Therefore, simultaneous horizontal transfer of transcription factors and their target genes might be necessary for successful horizontal transfer of regulatory genes.

### Importance of constraining factors in GRN and genomic evolution

Our study demonstrates that various constraining factors inherent in organisms could show significant impacts on GRN evolution. While redundant duplicated genes are common in various species, some microbial organisms, such as *Escherichia coli*, were known to have only a small number of duplicated genes and very few pseudo genes in their genome. This indicates that these organisms might show mutational biases toward high deletion rates or be living under the strong influence of selection for expression costs. Many studies have suggested that various biophysical factors involved in transcriptional regulations (e.g., molecular properties of DNA and proteins, physical structures of the nucleus and chromosomes, spatial arrangements of gene order in genomes, and stochastic noises of chemical reactions) would be important for the evolution of GRNs and genomic architectures [42]–[45], [77]–[79]. Thus, these biophysical constraints should be considered for the extension of the model.

We would like to emphasize the striking importance of considering constraining factors in evolutionary models to analyze the effect of selection on GRN and genomic evolution. Generally, many evolutionary biologists are often interested in questions such as how differences in genomes or GRNs among species are caused by selection or whether some characteristic properties of GRNs such as network motifs are evolved by selection or not. In the field of molecular evolution, evolutionary models have generally considered various mutational biases (e.g., base substitution model). In contrast, in the studies on biological network evolution, mathematical random network models have been usually used as null models to detect the effects of selection. Thus, previous studies on network evolution might be ineffective in detecting the effects of selection. To solve this problem, evolutionary models considering these constraining processes (e.g., mutation biases and biophysical factors) must be used as null models to detect the effects of selection.

### Multilateral aspects of mutational robustness and evolvability

Our study demonstrated that complex GRNs confer high mutational robustness (i.e., mutations against core genes are unlikely to cause *Loss-of-phenotype* and have only a small phenotypic effect) and evolvability (i.e., a larger mutational target size and a mutation are likely to change the phenotype) (Fig. 4). In contrast, simple GRNs confer only mutational robustness because of their small mutational target size. Increased core genes in complex GRNs are mostly functionally redundant duplicated genes in our model; thus, the proportion of mutations that cause *Loss-of-phenotype* seems to be small in complex GRNs. At the same time, an increase of redundant genes might reduce the contribution of each redundant gene to phenotypic expression; thus, the size of phenotypic change by *Significant* mutations might be small in complex GRNs. On the other hand, mutations against core genes generally unlikely to be *Non-effect*; thus, a decrease of *Loss-of-phenotype* mutations in complex GRNs leads to the increase of *Significant* mutations.

Many studies in evolutionary biology have studied the relationship between the mode of fluctuating selection and the evolution of mutational properties, such as genetic canalization and evolvability. Previous studies showed that genetic canalization (a kind of genetic robustness, which is defined as phenotypic insensitivity to mutation or a lower genetic variance of phenotype) would evolve under stabilizing selection and cyclically fluctuating selection with particularly small and frequent optimum shift [25], [71], [80]. Also, decanalization or higher evolvability would evolve under randomly fluctuating selection and cyclically fluctuating selection with large and infrequent optimum shifts [29], [39], [71]. In this study, simple GRNs evolved under conditions where genetic canalization is expected to evolve, while complex GRNs evolved under condition where decanalization is expected to evolve. Because a population continuously needs to follow in the movement of the optimum shift under selective conditions where decanalization was favored, the evolution of complex GRNs was promoted by phenotypic adaptation by gene duplication in these selective conditions.

The relationship between robustness and evolvability is a key to understanding how organisms can withstand mutations. However, multiple definitions of mutational robustness and evolvability made it difficult to understand the relationship and evolutionary origins of these features. For example, mutational robustness is defined as a property that reduces the phenotypic and lethal effects of mutations [25], while evolvability is defined as the ability to promote high evolution rates of an existing trait [11], [37] and the emergence of a novel trait [27]. These definitions of mutational robustness and evolvability indicate that they are interrelated with each other and include several distinct properties concerning the effect of mutations on phenotype and viability. Because robustness and evolvability are such complex traits, various mutational effects such as *Loss-of-phenotype*, *Non-effect*, and *Significant* should be considered to understand these mutational properties. For example, most quantitative traits in wild populations have substantial genetic variations (evolvability); however, systematic analysis of gene knockout experiments has revealed that mutations are unlikely to cause lethal outcomes and are likely to show only small phenotypic effects (mutational robustness) [81], [82]. However, the type of biological systems that could consistently achieve both mutational robustness and evolvability and the modes of environmental conditions by which such genetic systems could evolve remain unknown [83], [84]. Our study revealed that both mutational robustness and evolvability could be consistently achieved by complex GRNs that evolved under randomly fluctuating environments.

### Relationship between mutational robustness and genetic canalization

Genetic canalization has long been regarded as a proxy of mutational robustness in biology [80]. Thus, our results might be confusing since complex GRNs that evolved under the condition of decanalization have higher mutational robustness than simple GRNs that evolved under the condition of canalization in several aspects (mutations against core genes are unlikely to cause *Loss-of-phenotype* and are likely to cause only small phenotypic changes). In the studies on the evolution of genetic canalization with GRN [25], [85], genetic canalization was defined as a smaller average phenotypic effect of mutations. However, because these studies did not distinguish *Non-effect* and *Significant* mutations, it is not clear whether the smaller average phenotypic effect of mutations is due to the larger proportion of *Non-effect* mutation (i.e. high *P_N_*) or the smaller phenotypic effect of *Significant* mutations (i.e. small *D_S_*). Generally, some genes do not contribute to the expression of other genes when GRNs evolve under stabilizing selection, and these genes are called frozen components [86], [87]. If frozen components correspond to non-core genes in our model, mutations in frozen components mostly would not affect gene expression patterns of GRNs (i.e., *Non-effect*). Thus, the evolution of genetic canalization in previous GRN models showed robustness mainly due to larger proportion of *Non-effect* mutations rather than to smaller phenotypic effect of *Significant* mutations. In addition, these studies showed that evolution of genetic canalization in GRNs was associated with the evolution of shorter developmental time to establish a steady-state gene expression pattern [25], [85]. The results also indicated the evolution of simple GRNs (smaller number of core genes) in these models [87]. Additionally, Huerta-Sanchez and Durrett (2005) revealed that the evolution of genetic canalization in the model was due to the selection for increased viability against mutations rather than phenotypic selection [88]. In other words, selection for increased viability under pure stabilizing selection promotes smaller mutation rates of the phenotype (i.e. smaller mutational target size) rather than smaller phenotypic effects of mutations.

### Role of mutational robustness and evolvability in evolution

The importance of mutational robustness in evolution has been pointed out [41]. Robustness mechanisms generally would lessen the number of mutations that show deleterious effects and would increase the number of mutations that potentially contribute to phenotypic adaptation. Thus, robustness mechanisms are considered to have some effects that promote the evolvability of organisms in general. For example, in our analysis, the robustness conferred by redundant duplicated genes and other robustness mechanisms that reduce functional constraints that act on expression dynamics would lessen the proportion of *Loss-of-phenotype* mutations and increase the proportion of *Significant* mutations. Consequently, the rate of evolution in the existing trait (a kind of evolvability) is increased. Some studies argued that robustness mechanisms would lessen the number of mutations that show some phenotypic effects and would increase the number of neutral mutation. Moreover, these studies argued that the accumulation of such neutral mutations would aid the evolution of a novel phenotype (another definition of evolvability) when the environmental or genetic background was changed [83], [84]. Thus, the decrease of deleterious effects of mutations through some robustness mechanisms, including redundancy, Hsp proteins, posttranscriptional processes, and protein-protein interactions, might have some effects that can promote organismal evolvability in general.

Our results showed that the evolution of GRNs could occur when the effects of evolvability are absent (Fig. S2 and S3). The level of evolvability appeared to saturate at relatively small numbers of core genes (<10 or so) (Fig. S1). Because phenotypic selection strongly promoted the evolution of complex GRNs that had very large number of genes in our model, the effects of evolvability on GRN evolution might not be detected in our study. The level of complexity of a single regulatory module in real organisms is not so high; thus, the selection for evolvability might actually be effective for promoting complex GRNs. Our study did not analyze the effects of evolvability in GRNs with such small number of genes. Estimating the effects of evolvability alone on GRN evolution without the influence of phenotypic selection would be possible if the evolvability of each genotype is examined, and the genotype would be artificially selected according to their evolvability. Then, if evolution of complex GRNs is observed through the analysis, we can demonstrate that selection for evolvability alone could promote complex GRNs. However, such selective conditions might be unrealistic in nature, and selection for evolvability is inevitably coupled with phenotypic selection. Thus, it may be generally difficult to distinguish the effects of these two factors.

The role of evolvability in organismal evolution is an interesting subject in understanding the origin of biological diversity. Contrary to the ordinary phenotypes, evolvability is not a property of an individual. Instead, it is a property of a “genotype.” Because the evolvability of an original genotype itself would change by mutations, it should be applied only for short-term evolution. However, depending on the properties of target systems, e.g., very low gene duplication rates, the evolvability of an original genotype might be invariant to the mutations and might be applied even for long-term evolution. The mechanism by which properties of evolvability depend on the target systems will be an interesting subject in the future.

### Diverse effects of mutations on phenotypes and fitness

To analyze mutational robustness and evolvability, we used the phenotypic effects of mutations rather than fitness effects. This is because the phenotypic effects of mutations can be observed in laboratory experiments for real organisms, but the fitness effects of mutations differ depending on external environments and are very difficult to be measured. One of our aims in the present study was to clarify the multilateral aspects of mutational robustness and evolvability by using data available in laboratory experiments. While experimental noise would bring some difficulty in estimating the phenotypic effects, it would be possible to distinguish the effects of mutations such as *Loss-of-phenotype*, *Non-effect*, *Significant*, and also the mutations that change the expression dynamics through examining temporal changes of gene expression or variance of the expression. A distinction between *Significant* mutations and *Loss-of-phenotype* mutations in our analysis was actually helpful because the increased number of core genes mainly contributed to the increased number of *Significant* mutations and decreased number of *Loss-of-phenotype*, but minorly contribute to *Non-effect* mutations. Thus, it would be difficult to reveal the relationship between the structure and genetic properties of GRNs without distinguishing between *Significant* and *Loss-of-phenotype* mutations in our analysis. We believe that such a distinction between mutations is useful in understanding mutational robustness and evolvability.

Moreover, these diverse mutational effects might have fundamental importance in biological evolution. For example, some studies proposed that even mutations that were neutral at the time it arose (called cryptic genetic variation) might contribute to phenotypic adaptation because such cryptic genetic variations could contribute the phenotypic variation following changes of genetic and environmental background [89], [90]. In our simulations, we considered the unsteady dynamics of phenotypic gene expression as lethal. In addition, while the loss of phenotypic gene expression were not assumed to be lethal, the mutation was generally deleterious in our analysis (data not shown) because in our simulations, the movement of optimum was assumed to avoid around *P_i_* = 0. However, the mutations did not necessarily become lethal/deleterious in real organisms. For example, gene essentiality would be reduced under conditions where selective pressure might be weak, such as laboratory conditions or intrabody environments. Moreover, some studies have revealed that even loss of gene expression could be beneficial for phenotypic adaptation under a certain environment in nature [91]. In addition, unsteady expression dynamics might be favorable under fluctuating environments through its increased temporal variance of gene expressions. Recent technological advances have allowed us to perform not only whole genome expression analysis but also analysis of expression dynamics at the single-cell level [92]. By shifting the viewpoint regarding the effect of mutations from the changes in steady-state expression levels to the changes in expression dynamics, we could deal with broader aspects of GRN evolution and could understand organismal evolution in general.

### Future directions

Some predictions from our study might provide useful hypotheses that can be tested by experimental data in real organisms. For example, Roth (1989) conducted an experiment on microbial evolution and showed that a duplication-containing strain was fixed under conditions of growth limitation because of the availability of a carbon and energy source, but the strain was displaced by other strains afterward [93]. Experimental evolution under unexpectedly fluctuating environments might promote increasing number of gene duplication.

The present models did not consider several important factors such as duplication of receptor and phenotypic genes, stochastic noise, pleiotropy, and complexity of gene regulation. Duplication and divergence of receptors and target genes are commonly observed in microbial GRN evolution [94]. Extending our models would make it possible to study GRN evolution in broader contexts, such as evolution of new functions, adaptation to novel environment, and evolution of complex phenotypes. Many studies have revealed that even simple genetic networks could show robustness against the noise of gene expressions by means of some local network architectures called network motifs [95]–[98]. Examining how gene expression noise affects the evolution of GRNs under various fluctuating selection conditions would be interesting. In multicellular organisms, many transcription factors work at several developmental stages or in multiple cell types. The pleiotropic property of genes would be necessary for GRNs of multicellular organisms. Molecular interactions between the DNA, transcription factors, and transcription machinery are extremely complex. While simulating all of these interactions is impossible, considering some interactions is necessary to explore their importance. A sequence-based GRN model that considers molecular interactions might aid in detailed quantitative analyses of the evolution of gene expression and GRNs [99].

## Methods

### Structures of genes and GRNs

The GRN of each individual had *M* phenotypic genes and *N* regulatory genes. Each gene was composed of a *cis*-regulatory region and a coding region. A *cis*-regulatory region was composed of *L cis*-sites that are potentially recognized by specific transcription factors (boxes in Fig. 1A), and each *cis*-site had two parameters called the *cis*-number and the interaction coefficient. On the other hand, a coding region (diamonds in Fig. 1A) had a parameter called the *trans*-number. The *cis*- and *trans*-number values determined which regulatory gene product (i.e., transcription factor) would bind to a *cis*-site. For example, the product of a regulatory gene with a *trans*-number of 5 would bind to *cis*-sites with a *cis*-number of 5 (see Fig. S9 for an illustration). The value of the interaction coefficient determines the strength of transcriptional activation/repression when a regulatory gene product binds to the *cis*-site. A *cis*- and *trans*-number was assigned an integral number in the range [1, *n*]. An interaction coefficient had a real value in the range [−5, 5].

A *cis*-number represented a specific DNA sequence of *m* base pairs. The possible number of motifs (*n*; the possible number of colors in Fig. 1A) produced by *m* base pairs of DNA sequence was calculated as:
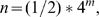
(2)where 1/2 indicates the direction of motifs against the promoter. Multiple binding sites for the same transcription factor were allowed to exist in a *cis*-regulatory region. However, not all the *cis*-sites were bounded by transcription factors because the possible number of motifs (*n*) was much greater than the number of regulatory genes that actually existed in a genome (*N*); *n*≫*N*. Generally, the length of DNA sequences that were recognized by a transcription factor (*m*) was 5–10 bp; thus, we assumed *m*≈7.14 for all the regulatory genes (this corresponded to *n* = 9950).

### Dynamics of gene expression and phenotype

A GRN was represented by a dynamic system whose state was represented by the expression levels of the network genes, which were denoted as:
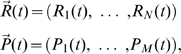
(3)where *R_i_*(*t*) and *P_i_*(*t*) are the expression levels of the regulatory gene *i* and phenotypic gene *i* at developmental time *t*, respectively. The gene expression state at *t* = 0 is the initial gene expression state. The initial gene expression state for all genes was set at the 0.0 expression level. Thus, the initial gene expression state was represented as:
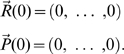
(4)Certain genes were assumed to have a positive basal transcription level (described below), and these genes began to express without transcriptional activation by regulatory genes soon after the beginning of development. The expression level of each gene would change by the following equation:
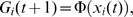
(5)where *G_i_*(*t*) is the expression level of gene *i* (*R_i_* or *P_i_*) and *x_i_*(*t*) is the regulatory input to gene *i* at developmental time *t*. The Φ value was defined by:
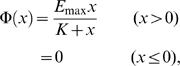
(6)where *K* and *E*_max_ was constant that determines threshold against regulatory input and the maximum gene expression level and was set at 15 and 10 for all the genes, respectively. This value restricted the expression level to a range [0.0, 10.0] for all genes. The regulatory input to gene *i* at developmental time *t* was calculated as:
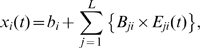
(7)where *b_i_* is the basal transcription level of gene *i* (*b_i_*≥0), *L* is the number of *cis*-sites, *B_ji_* is the interaction coefficient of the *cis*-site *j* of gene *i*, and *E_ji_*(*t*) is the sum of the expression levels of all the regulatory genes that bind to the *cis*-site *j* of gene *i* at developmental time *t*. We assumed that half of the genes in a GRN at generation 0 had *b* = 1, while the other half of genes had *b* = 0.

We considered the equilibrium steady-state expression levels of phenotypic genes as individual phenotype, which was described as:
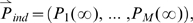
(8)The steady state was achieved when the following variance-like criterion was met for all the phenotypic genes
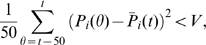
(9)where 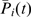 is the mean expression level of the phenotypic gene *i* over the developmental time from (*t*−50) to *t*, and *V* determined the degree of steady-state levels that were required for the viable phenotypic expression (*V* = 10^−4^ for standard parameter values). In addition, an individual that did not reach the steady state within the developmental time of 500 was considered to be lethal.

For the modeling of external signals, we assumed that the *R*_1_ gene was a receptor transcription factor. *R_1_* can exist either in the active state (*R*_1_^+^), which can control transcription, or in the inactive state (*R*_1_^−^), which cannot control transcription. If external signals were present, all products of the *R*_1_ gene stayed active throughout the developmental process; however, if the external signals were absent, all products of the *R*_1_ gene stayed inactive throughout. Thus, an individual had two phenotypic states: 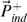 (in the presence of an external signal) and 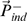 (in the absence of an external signal).

### Fitness

The fitness value of an individual (*F*) was calculated by
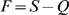
(10)where *S* is the suitability of the individual's phenotype to the environmental conditions, and *Q* is the cost of expressing the phenotype. The suitability of phenotype (*S*) was determined by the following Gaussian function:
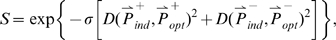
(11)where *D* is the Euclidean distance between the phenotype of an individual (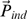) and the optimal phenotype (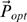), *σ* represents the strength of phenotypic selection (*σ* = 1), and 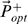 or 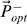 is the optimal phenotype in the presence or absence of an external signal, respectively. Because expressing phenotypic genes in the absence of external signals would be wasteful, we assumed 
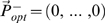
 for all simulation conditions. On the other hand, the state 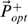 was assumed to change temporally, as described in the main text, according to fluctuations in external conditions. The cost of expressing the phenotype (*Q*) was described as:
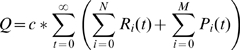
(12)where *c* is the fitness load per unit of gene expression and *R_i_*(*t*) and *P_i_*(*t*) are the expression levels of the *R* and *P* genes *i* at developmental time *t*, respectively (*c* = 10^−5^ for standard parameter values).

Then, the probability of reproduction (*W_i_*) that a copy (i.e., offspring) of individual *i* was created for the next generation was described as:
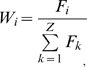
(13)where *Z* is the effective population size (*Z* = 10^5^). Thus, in creating the next generation, one individual was selected according to the probability, and this procedure was repeated until we got *Z* viable offspring.

### Mutation

When a copy of an individual (offspring) was created, mutation would occur at a certain probability. Six types of mutations (gene duplication, gene deletion, *cis*-regulatory mutation, trans-regulatory mutation, basal transcription level mutation, and horizontal gene transfer) were assumed in the model, and the per-gene mutation rates for each type of mutations were denoted as *μ_BTL_*, *μ_CIS_*, *μ_TRA_*, *μ_DUP_*, *μ_DEL_*, and *μ_HOR_*, respectively. When a gene duplication (or gene deletion) was assumed to occur, one regulatory gene was randomly copied (or erased) along with its *cis*-regulatory and coding regions. When a *cis*-regulatory mutation (or *trans*-regulatory mutation) was assumed to occur, a *cis*-site (or the coding region) was randomly chosen and the value of its *cis*-number (or *trans*-number) was replaced by the value drawn from the uniform distribution of the integer [1, *n*]. When a basal transcription level mutation was assumed to occur, a regulatory or phenotypic gene was randomly chosen, and the value of the basal transcription level of the gene (*b*) was increased (+1) or decreased (−1). When a horizontal gene transfer was assumed to occur, a regulatory gene was randomly created by assigning values drawn from uniformly distributed integer [1, *n*] to each *cis*- and *trans*-number, and by assigning values drawn from uniformly distributed real number [−5, +5]. *R*_1_ is the receptor for upstream signals; thus, duplication and deletion were not assumed to occur in this gene.

Although the per-gene mutation rate of the *cis*-regulatory region was not estimated, based on the mutation rate per nucleotide per generation (10^−10^) and the extent of the *cis*-regulatory region of genes (10^2^–10^5^ bp) [50], [100], the mutation rate including the *cis*-regulatory region would be in the order of 10^−8^–10^−5^ per gene per generation. Background rates of gene duplication and deletion are approximated in the order of 10^−6^ per gene per cell division (generation) in yeast; on the other hand, per-generation mutation rates in multicellular organisms could be 1 to nearly 3 orders of magnitude greater than that in yeast because germ-line cell divisions occur 9 times in nematodes, 36 times in flies, and 200 times in humans [49]. Therefore, the mutation rates used in this study are probably realistic.

### Per-individual mutation rate

Because offspring are assumed to be subject to a mutation at per-individual mutation rate regardless of the number of genes, only a single mutation was always introduced to the offspring. The per-individual rate of each mutation was set at values 10 times larger than the per-gene rates because a founder individual has 10 regulatory genes.

### Constant *P_S_*

For setting *P_S_* = 1 and *P_N_* = *P_L_* = 0, we reintroduced a mutation to the original offspring until the mutation showed *Significant* phenotypic changes when the offspring were subjected to a mutation. Notably, this procedure did not cause multiple mutations in the offspring. We assumed *P_S_* = 1 only for gene duplication, gene deletion and trans-regulatory mutation, and the other type of mutations are assumed as same as the original model. Because, the value of *P_S_* in gene duplications, gene deletions and trans-regulatory mutations were well correlated to the structure of GRNs. On the other hand, cis-regulatory mutations and basal transcription level mutations originally had very low value of *P_S_*, and the procedure of setting *P_S_* = 1 in these mutations would disrupt the evolution of GRNs in unwilling manner.

### Measurement of phenotypic adaptation rate

To examine the rate of phenotypic adaptation of an evolved population, a new optimum was placed at a distance away (d = 1) from the mean phenotype of the population. Then, under this benchmark selective condition, the population was allowed to evolve for 1000 generations. During the benchmark evolution, changes in the Euclidean distance between the optimum and the mean phenotype of populations were examined.

### Definition of *C_mut_*

The probability that a binding site of a particular transcription factor is present in a *cis*-regulatory region depended on the size (base pair) of the binding site (*m*) and the *cis*-regulatory region (*L*) [70]. The number of DNA motifs (*n*) produced by *m* base pairs of DNA sequences was calculated as *n* = (1/2)×4*^m^* as described by Equation 2. Thus, the probability of the presence (*C_mut_*) and absence (1−*C_mut_*) of a binding site of a particular transcription factor in a *cis*-regulatory region was represented by the following equation (see Fig. S9 for an illustration).
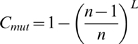
(14)where *L* is the number of *cis*-sites (i.e., potential binding sites) in a *cis*-regulatory region. The value of *L* was changed to control the value of *C_mut_* in the simulation. For the standard parameter values, we used *L* = 100, *n* = 9550 (*m* = 7.14), and, thus, *C_mut_*≈0.01. This probability was applied to all combinations of any transcription factor and any *cis*-regulatory region in a GRN; therefore, the value of the GRN connectivity density (*C*; the proportion of the number of existing regulatory interactions against the number of possible regulatory interactions in a GRN) tended to approach the value of *C_mut_* if sufficient numbers of regulatory mutation were accumulated. To represent the relative rate of gain and loss of regulatory interactions, Lynch (2007) used a different parameter, *α* = *μ_l_*/*μ_g_*, where *μ_l_* and *μ_g_* are the rate of loss and gain of a transcription factor binding sites, respectively [31]. We approximate that *α* = *μ_l_*/*μ_g_*≈(1−*C_mut_*)/*C_mut_*. Lynch (2007) inferred *α* = 10^3^−10^2^ for prokaryotes, 10^2^−10^1^ for unicellular eukaryotes, and 10^1^−10^0^ for multicellular eukaryotes; this corresponds to *C_mut_* = 10^−3^–10^−2^ for prokaryotes, 10^−2^–10^−1^ for unicellular eukaryotes, and 10^−1^–10^0^ for multicellular eukaryotes.

### Preparation of the initial population

To prepare the initial population, we created a founder individual for each population. A founder individual had *M_ini_* phenotypic genes and *N_ini_* regulatory genes; its GRN structure was randomly generated with a certain connectivity (*C_init_*). We used *M_ini_* = 10, *N_ini_* = 2, and *C_init_* = 0.5 for the standard simulation condition because the low value of *C_init_* made it difficult to obtain viable founder individuals. Qualitatively similar results were obtained with the various values of *M_ini_*, *N_ini_* (Fig. S7 and S8), and *C_init_*. We assumed *b* = 1 for half of the genes and *b* = 0 for the other half. To create a founder individual with a certain value of *C_init_*, we determined the range of values for *cis*- and *trans*-numbers (*n_init_*) according to Equation 14 (*L* = 100, standard parameter value; thus, *n_init_* = 145 for *C_init_* = 0.5). We then initialized the genome of the founder individual by setting the random integral number between 1 and *n_init_* for each *cis*- and *trans*-number, and the random real number between −5 and +5 for each interaction coefficient. This procedure assured that the GRN connectivity (*C*) of the founder individual equaled to *C_init_*. Then, the *cis*-numbers that were not used for regulatory interactions were rerandomized by setting a random integral number ranging [1, n]; however, the numbers that were already assigned as *trans*-numbers were excluded from the rerandomization. This procedure assured the sufficient complexity of the composition of the *cis*-regulatory regions of the founding individual. We assumed that the viable founder individual should have appropriate phenotypic values, in which the expression levels of all phenotypic genes are <0.01 in 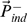 and >2.0 in 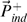.

## Supporting Information

Figure S1 Relationship between the rate of phenotypic adaptation and the properties of GRNs. After 50,000 generations in the experimental evolution, a new optimum was placed at a constant distance away (*d* = 1) from the mean phenotype of the population. The population was then allowed to evolve for 1000 generations (denoted as the benchmark evolution). Points represent the results of each population in the random-walk optimum shift. Horizontal axes indicate the number of core genes and *P_S_* of a population at the end of experimental evolution. *D_i_* indicates the Euclidean distance between the optimum and the mean phenotype of a population at generation *i* during the benchmark evolution. Kendall's correlation test was used for statistical analysis of the correlation.
(0.31 MB TIF)

Figure S2 Number of regulatory genes in GRN that evolved under fixed per-individual mutation rate. GRNs were allowed to evolve with a fixed per-individual mutation rate regardless of the number of genes in GRNs. *μ_BTL_* = *μ_CIS_* = *μ_TRA_* = *μ_DEL_* = *μ_DUP_* = 10^−5^ per-individual per generation. Each point connected by solid lines represents the mean number of each type of genes in evolved GRNs under each selective condition. Vertical bars attached to the point represent 95% confidence intervals. *d* and *f* represent the amplitude and frequency of the optimum shift, respectively.
(0.16 MB TIF)

Figure S3 Number of regulatory genes in GRN that evolved under constant P_S_. GRNs were allowed to evolve with a constant P_S_ regardless of the number of genes in GRNs (P_S_ = 1, P_L_ = P_N_ = 0). *μ_BTL_* = *μ_CIS_* = *μ_TRA_* = *μ_DEL_* = *μ_DUP_* = 10^−5^ per-individual per generation. Each point connected by solid lines represents the mean number of each type of genes in evolved GRNs under each selective condition. Vertical bars attached to the point represent 95% confidence intervals. *d* and *f* represent the amplitude and frequency of the optimum shift, respectively.
(0.16 MB TIF)

Figure S4 Number of regulatory genes in GRN that evolved under various rates of cis- and trans-regulatory mutations (*μ_CIS_*, *μ_TRA_*). The values of both *μ_CIS_* and *μ_TRA_* are varied from 10^−8^ to 10^−6^ (*μ_CIS_* = *μ_TRA_* = 10^−6^, standard parameter value). Points connected by solid lines represent the mean number of core genes (#core), pseudo-expression genes (#psdexp), and silent genes (#silent) in GRNs that evolved for 50,000 generations under each simulation condition. Vertical bars indicate 95% confidence intervals. Different colors indicate different conditions of phenotypic selection; d = 10^−1^, f = 10^−1^ (red); d = 10^0^, f = 10^−3^ (blue); d = 10^−3^, f = 10^−3^ (black) under random-walk optimum shift.
(0.13 MB TIF)

Figure S5 Indegree distribution of assembled GRNs that evolved under various rates of cis- and trans-regulatory mutations (*μ_CIS_*, *μ_TRA_*). The values of both *μ_CIS_* and *μ_TRA_* are varied from 10^−8^ to 10^−6^, standard parameter value). Horizontal and vertical axes in each panel show the indegree (the number of regulatory interactions that arrived at a gene) and the frequency, respectively. Note that the vertical axes are shown logarithmically to demonstrate the exponential character of the distribution. Different rows and columns show the indegree distributions of GRNs under different conditions of phenotypic selection and different values of (*μ_CIS_*, *μ_TRA_*), respectively. Lines in each panel indicate the regression of the plot to the Power law distribution (red), the exponential distribution (blue) and the Poisson distribution (green). Regression was estimated by a nonlinear least-square method. To judge the goodness of the regression, Akaike's information criterion (AIC) was used, and the regression that showed the smallest value of AIC was drawn as a thick line. POW, EXP and POI in each panel indicate the differences between AIC value of the best regression model and those of power-law (scale-free), exponential and poisson distributions, respectively.
(0.52 MB TIF)

Figure S6 Outdegree distribution of assembled GRNs that evolved under various rates of cis- and trans-regulatory mutations (*μ_CIS_*, *μ_TRA_*). The values of both *μ_CIS_* and *μ_TRA_* are varied from 10^−8^ to 10^−6^ (*μ_CIS_* = *μ_TRA_* = 10^−6^, the standard parameter value). Horizontal and vertical axes in each panel show the outdegree (the number of regulatory interactions that depart from a gene) and the frequency, respectively. Note that the both horizontal and vertical axes are shown logarithmically to demonstrate the scale-free character of the distribution. Different rows and columns show the outdegree distributions of GRNs under different conditions of phenotypic selection and different values of (*μ_CIS_*, *μ_TRA_*), respectively. Lines in each panel indicate the regression of the plot to the Power law distribution (red), exponential distribution (blue) and Poisson distribution (green). Regression was estimated by a nonlinear least-square method. To judge the goodness of the regression, Akaike's information criterion (AIC) was used, and the regression that showed the smallest value of AIC was drawn as a thick line. POW, EXP and POI in each panel indicate the differences between AIC value of the best regression model and those of power-law (scale-free), exponential and poisson distributions, respectively.
(0.55 MB TIF)

Figure S7 Number of regulatory genes in GRN that evolved under the various initial numbers of regulatory genes (*N_init_*). *N_init_* = 10, standard parameter value. Points connected by solid lines represent the mean number of core genes (#core), pseudo-expression genes (#psdexp), and silent genes (#silent) in GRNs that evolved for 50,000 generations under each simulation condition, respectively. Vertical bars indicate 95% confidence intervals. Different colors indicate the different conditions of phenotypic selection; d = 10^−1^, f = 10^−1^ (red); d = 10^0^, f = 10^−3^ (blue); d = 10^−3^, f = 10^−3^ (black) under random-walk optimum shift.
(0.13 MB TIF)

Figure S8 Number of regulatory genes in GRN that evolved under various initial numbers of phenotypic genes (*M_init_*). *M_init_* = 2, standard parameter value. Points connected by solid lines represent the mean number of core genes (#core), pseudo-expression genes (#psdexp), and silent genes (#silent) in GRNs that evolved for 50,000 generations under each simulation condition, respectively. Vertical bars indicate 95% confidence intervals. Different colors indicate the different conditions of phenotypic selection; d = 10^−1^, f = 10^−1^ (red); d = 10^0^, f = 10^−3^ (blue); d = 10^−3^, f = 10^−3^ (black) under random-walk optimum shift.
(0.11 MB TIF)

Figure S9 Relationship between gene structures and *C_mut_*.
(0.14 MB TIF)

Figure S10 Illustration of the relationship between the intensity of optimum fluctuation and the fitness effects of a certain mutation.
(0.09 MB TIF)

## References

[1] Carroll SB (2008). Evo-devo and an expanding evolutionary synthesis: a genetic theory of morphological evolution.. Cell.

[2] Fox CW, Wolf JB (2006). Evolutionary genetics : concepts and case studies.

[3] Prud'homme B, Gompel N, Carroll SB (2007). Emerging principles of regulatory evolution.. Proc Natl Acad Sci USA.

[4] Janga SC, Collado-Vides J (2007). Structure and evolution of gene regulatory networks in microbial genomes.. Res Microbiol.

[5] Lozada-Chávez I, Janga SC, Collado-Vides J (2006). Bacterial regulatory networks are extremely flexible in evolution.. Nucleic Acids Res.

[6] Babu MM, Teichmann SA, Aravind L (2006). Evolutionary dynamics of prokaryotic transcriptional regulatory networks.. J Mol Biol.

[7] McAdams HH, Srinivasan B, Arkin AP (2004). The evolution of genetic regulatory systems in bacteria.. Nat Rev Genet.

[8] Kopp M, Hermisson J (2007). Adaptation of a quantitative trait to a moving optimum.. Genetics.

[9] Lynch M (2007). The origins of genome architecture.

[10] Kirschner M, Gerhart J (1998). Evolvability.. Proc Natl Acad Sci USA.

[11] Landry CR, Lemos B, Rifkin SA, Dickinson WJ, Hartl DL (2007). Genetic properties influencing the evolvability of gene expression.. Science.

[12] Psujek S, Beer RD (2008). Developmental bias in evolution: evolutionary accessibility of phenotypes in a model evo-devo system.. Evol Dev.

[13] Wagner GP, Altenberg L (1996). Perspective: Complex adaptations and the evolution of evolvability.. Evolution.

[14] Arthur W (2004). The effect of development on the direction of evolution: toward a twenty-first century consensus.. Evol Dev.

[15] Félix M-A, Barrière A (2005). Evolvability of cell specification mechanisms.. J Exp Zool B Mol Dev Evol.

[16] Wilkins AS (2002). The Evolution of Developmental Pathways.

[17] Farkas IJ, Wu C, Chennubhotla C, Bahar I, Oltvai ZN (2006). Topological basis of signal integration in the transcriptional-regulatory network of the yeast, Saccharomyces cerevisiae.. BMC Bioinfo.

[18] Teichmann SA, Babu MM (2004). Gene regulatory network growth by duplication.. Nat Genet.

[19] Bhan A, Galas DJ, Dewey TG (2002). A Duplication Growth Model of Gene Expression Networks.. Bioinformatics.

[20] Lagomarsino MC, Jona P, Bassetti B, Isambert H (2007). Hierarchy and feedback in the evolution of the Escherichia coli transcription network.. Proc Natl Acad Sci USA.

[21] Guelzim N, Bottani S, Bourgine P, Kepes F (2002). Topological and Causal Structure of the Yeast Transcriptional Regulatory Network.. Nat Genet.

[22] Babu MM, Luscombe NM, Aravind L, Gerstein M, Teichmann SA (2004). Structure and evolution of transcriptional regulatory networks.. Curr Opin Struct Biol.

[23] Klemm K, Bornholdt S (2005). Topology of biological networks and reliability of information processing.. Proc Natl Acad Sci USA.

[24] Yu H, Gerstein M (2006). Genomic analysis of the hierarchical structure of regulatory networks.. Proc Natl Acad Sci USA.

[25] Siegal ML, Bergman A (2002). Waddington's canalization revisited: Developmental stability and evolution.. Proc Natl Acad Sci USA.

[26] Gu Z, Steinmetz LM, Gu X, Scharfe C, Davis RW (2003). Role of duplicate genes in genetic robustness against null mutations.. Nature.

[27] Aldana M, Balleza E, Kauffman SA, Resendiz O (2007). Robustness and evolvability in genetic regulatory networks.. J Theor Biol.

[28] Siegal ML, Promislow DEL, Bergman A (2007). Functional and evolutionary inference in gene networks: does topology matter?. Genetics.

[29] Crombach A, Hogeweg P (2008). Evolution of evolvability in gene regulatory networks.. PLoS Comput Biol.

[30] McShea D (2005). The evolution of complexity without natural selection, a possible large-scale trend of the fourth kind.. Paleobiol.

[31] Lynch M (2007). The evolution of genetic networks by non-adaptive processes.. Nat Rev Genet.

[32] Lynch M (2007). The frailty of adaptive hypotheses for the origins of organismal complexity.. Proc Natl Acad Sci USA.

[33] Félix M-A, Wagner A (2008). Robustness and evolution: concepts, insights and challenges from a developmental model system.. Heredity.

[34] Noort Vv, Snel B, Huynen MA (2004). The Yeast Coexpression Network has a Small-World, Scale-Free Architecture and can be Explained by a Simple Model.. EMBO Rep.

[35] Gu X (2003). Evolution of duplicate genes versus genetic robustness against null mutations.. Trends in Genetics.

[36] Sung H-M, Wang T-Y, Wang D, Huang Y-S, Wu J-P (2009). Roles of trans and cis variation in yeast intraspecies evolution of gene expression.. Mol Biol Evol.

[37] Carter AJ, Hermisson J, Hansen TF (2005). The role of epistatic gene interactions in the response to selection and the evolution of evolvability.. Theor Popul Biol.

[38] Earl DJ, Deem MW (2004). Evolvability is a selectable trait.. Proc Natl Acad Sci USA.

[39] Kashtan N, Noor E, Alon U (2007). Varying environments can speed up evolution.. Proc Natl Acad Sci USA.

[40] Ciliberti S, Martin OC, Wagner A (2007). Robustness can evolve gradually in complex regulatory gene networks with varying topology.. PLoS Comput Biol.

[41] Wagner A (2007).

[42] Janga SC, Collado-Vides J, Babu MM (2008). Transcriptional regulation constrains the organization of genes on eukaryotic chromosomes.. Proc Natl Acad Sci USA.

[43] Lozada-Chavez I, Angarica VE, Collado-Vides J, Contreras-Moreira B (2008). The role of DNA-binding specificity in the evolution of bacterial regulatory networks.. J Mol Biol.

[44] Janga S, Salgado H, Martínez-Antonio A (2009). Transcriptional regulation shapes the organization of genes on bacterial chromosomes.. Nucleic Acids Res.

[45] Kolesov G, Wunderlich Z, Laikova ON, Gelfand MS, Mirny LA (2007). How gene order is influenced by the biophysics of transcription regulation.. Proc Natl Acad Sci USA.

[46] Berg J, Willmann S, Lassig M (2004). Adaptive evolution of transcription factor binding sites.. BMC Evol Biol.

[47] Kondrashov FA, Kondrashov AS (2006). Role of Selection in Fixation of Gene Duplications.. J Theor Biol.

[48] Hooper SD, Berg OG (2003). On the nature of gene innovation: duplication patterns in microbial genomes.. Mol Biol Evol.

[49] Lynch M, Sung W, Morris K, Coffey N, Landry CR (2008). A genome-wide view of the spectrum of spontaneous mutations in yeast.. Proc Natl Acad Sci USA.

[50] Alon U (2006). An introduction to systems biology: design principles of biological circuits.

[51] Dekel E, Alon U (2005). Optimality and evolutionary tuning of the expression level of a protein.. Nature.

[52] Wagner A (2005). Energy constraints on the evolution of gene expression.. Mol Biol Evol.

[53] Bragg JG, Wagner A (2009). Protein material costs: single atoms can make an evolutionary difference.. Trends Genet.

[54] Zheng D, Gerstein M (2007). The ambiguous boundary between genes and pseudogenes: the dead rise up, or do they?. Trends in Genetics.

[55] Frith MC, Wilming LG, Forrest A, Kawaji H, Tan SL (2006). Pseudo-messenger RNA: Phantoms of the transcriptome.. PLoS Genet.

[56] Hanada K, Zou C, Lehti-Shiu MD, Shinozaki K, Shiu S-H (2008). Importance of lineage-specific expansion of plant tandem duplicates in the adaptive response to environmental stimuli.. Plant Physiol.

[57] Makino T, McLysaght A (2010). Ohnologs in the human genome are dosage balanced and frequently associated with disease.. Proc Natl Acad Sci U S A.

[58] Gompel N, Prud'homme B, Wittkopp PJ, Kassner VA, Carroll SB (2005). Chance Caught on the Wing: cis-Regulatory Evolution and the Origin of Pigment Patterns in Drosophila.. Nature.

[59] Huang S, Eichler G, Bar-Yam Y, Ingber DE (2005). Cell fates as high-dimensional attractor states of a complex gene regulatory network.. Phys Rev Lett.

[60] Arthur W (2000). The concept of developmental reprogramming and the quest for an inclusive theory of evolutionary mechanisms.. Evol Dev.

[61] Shapiro MD, Marks ME, Peichel CL, Blackman BK, Nereng KS (2004). Genetic and developmental basis of evolutionary pelvic reduction in threespine sticklebacks.. Nature.

[62] Abzhanov A, Kuo WP, Hartmann C, Grant BR, Grant PR (2006). The calmodulin pathway and evolution of elongated beak morphology in Darwin's finches.. Nature.

[63] Oleksiak MF, Roach JL, Crawford DL (2005). Natural variation in cardiac metabolism and gene expression in Fundulus heteroclitus.. Nat Genet.

[64] Babu MM, Aravind L (2006). Adaptive evolution by optimizing expression levels in different environments.. Trends Microbiol.

[65] Orr HA (2005). The genetic theory of adaptation: a brief history.. Nat Rev Genet.

[66] López-Maury L, Marguerat S, Bähler J (2008). Tuning gene expression to changing environments: from rapid responses to evolutionary adaptation.. Nat Rev Genet.

[67] Luscombe NM, Babu MM, Yu H, Snyder M, Teichmann SA (2004). Genomic analysis of regulatory network dynamics reveals large topological changes.. Nature.

[68] Balazsi G, Barabasi AL, Oltvai ZN (2005). Topological units of environmental signal processing in the transcriptional regulatory network of Escherichia coli.. Proc Natl Acad Sci USA.

[69] Ma HW, Buer J, Zeng AP (2004). Hierarchical Structure and Modules in the Escherichia Coli Transcriptional Regulatory Network Revealed by a new top-Down Approach.. BMC Bioinfo.

[70] Hansen TF, Álvarez-Castro JM, Carter AJR, Hermisson J, Wagner GP (2006). Evolution of Genetic Architecture under Directional Selection.. Evolution.

[71] Kawecki TJ (2000). The evolution of genetic canalization under fluctuating selection.. Evolution.

[72] Hunt G (2007). The relative importance of directional change, random walks, and stasis in the evolution of fossil lineages.. Proc Natl Acad Sci USA.

[73] Soyer OS, Bonhoeffer S (2006). Evolution of complexity in signaling pathways.. Proc Natl Acad Sci USA.

[74] Freeman JL, Perry GH, Feuk L, Redon R, McCarroll SA (2006). Copy number variation: New insights in genome diversity.. Genome Res.

[75] Stranger BE, Forrest MS, Dunning M, Ingle CE, Beazley C (2007). Relative Impact of Nucleotide and Copy Number Variation on Gene Expression Phenotypes.. Science.

[76] Hurst LD, Pál C, Lercher MJ (2004). The evolutionary dynamics of eukaryotic gene order.. Nat Rev Genet.

[77] Huerta AM, Francino MP, Morett E, Collado-Vides J (2006). Selection for unequal densities of sigma70 promoter-like signals in different regions of large bacterial genomes.. PLoS Genet.

[78] Janga SC, Salgado H, Martínez-Antonio A, Collado-Vides J (2007). Coordination logic of the sensing machinery in the transcriptional regulatory network of Escherichia coli.. Nucleic Acids Res.

[79] Martínez-Antonio A, Janga SC, Thieffry D (2008). Functional organisation of Escherichia coli transcriptional regulatory network.. J Mol Biol.

[80] de Visser JAGM, Hermisson J, Wagner GP, Ancel Meyers L, Bagheri-Chaichian H (2003). Perspective: Evolution and detection of genetic robustness.. Evolution.

[81] Giaever G, Chu AM, Ni L, Connelly C, Riles L (2002). Functional profiling of the Saccharomyces cerevisiae genome.. Nature.

[82] Kamath R, Fraser A, Dong Y, Poulin G, Durbin R (2003). Systematic functional analysis of the Caenorhabditis elegans genome using RNAi.. Nature.

[83] Wagner A (2008). Robustness and evolvability: a paradox resolved.. Proc Biol Sci.

[84] Ciliberti S, Martin O, Wagner A (2007). Innovation and robustness in complex regulatory gene networks.. Proc Natl Acad Sci USA.

[85] Wagner A (1996). Does Evolutionary Plasticity Evolve?. Evolution.

[86] Kauffman SA (1990). Requirements for evolvability in complex-systems - orderly dynamics and frozen components.. Physica D.

[87] Bornholdt S (2001). Modeling genetic networks and their evolution: A complex dynamical systems perspective.. Biol Chem.

[88] Huerta-Sanchez E, Durrett R (2007). Wagner's canalization model.. Theor Popul Biol.

[89] Le Rouzic A, Carlborg O (2008). Evolutionary potential of hidden genetic variation.. Trends Ecol Evol.

[90] Schlichting CD (2008). Hidden reaction norms, cryptic genetic variation, and evolvability.. Ann N Y Acad Sci.

[91] Chan YF, Marks ME, Jones FC, Villarreal G, Shapiro MD (2010). Adaptive evolution of pelvic reduction in sticklebacks by recurrent deletion of a Pitx1 enhancer.. Science.

[92] Bennett MR, Hasty J (2009). Microfluidic devices for measuring gene network dynamics in single cells.. Nat Rev Genet.

[93] Sonti RV, Roth JR (1989). Role of Gene Duplications in the Adaptation of Salmonella Typhimurium to Growth on Limiting Carbon Sources.. Genetics.

[94] Maslov S, Krishna S, Pang TY, Sneppen K (2009). Toolbox model of evolution of prokaryotic metabolic networks and their regulation.. Proceedings of the National Academy of Sciences of the United States of America.

[95] Kaern M, Elston TC, Blake WJ, Collins JJ (2005). Stochasticity in gene expression: from theories to phenotypes.. Nat Rev Genet.

[96] Becskei A, Serrano L (2000). Engineering Stability in Gene Networks by Autoregulation.. Nature.

[97] Kalir S, Mangan S, Alon U (2005). A coherent feed-forward loop with a SUM input function prolongs flagella expression in Escherichia coli.. Mol Syst Biol.

[98] Mangan S, Zaslaver A, Alon U (2003). The coherent feedforward loop serves as a sign-sensitive delay element in transcription networks.. J Mol Biol.

[99] Segal E, Widom J (2009). From DNA sequence to transcriptional behaviour: a quantitative approach.. Nat Rev Genet.

[100] Wray GA, Hahn MW, Abouheif E, Balhoff JP, Pizer M (2003). The Evolution of Transcriptional Regulation in Eukaryotes.. Mol Biol Evol.

