# Supplementary figures and images for: Evolution of Gene Regulatory Networks by Fluctuating Selection and Intrinsic Constraints
Source: PLoS Comput Biol. 2010 Aug 5;6(8):e1000873. doi: 10.1371/journal.pcbi.1000873 (PMC2916849; doi:10.1371/journal.pcbi.1000873)

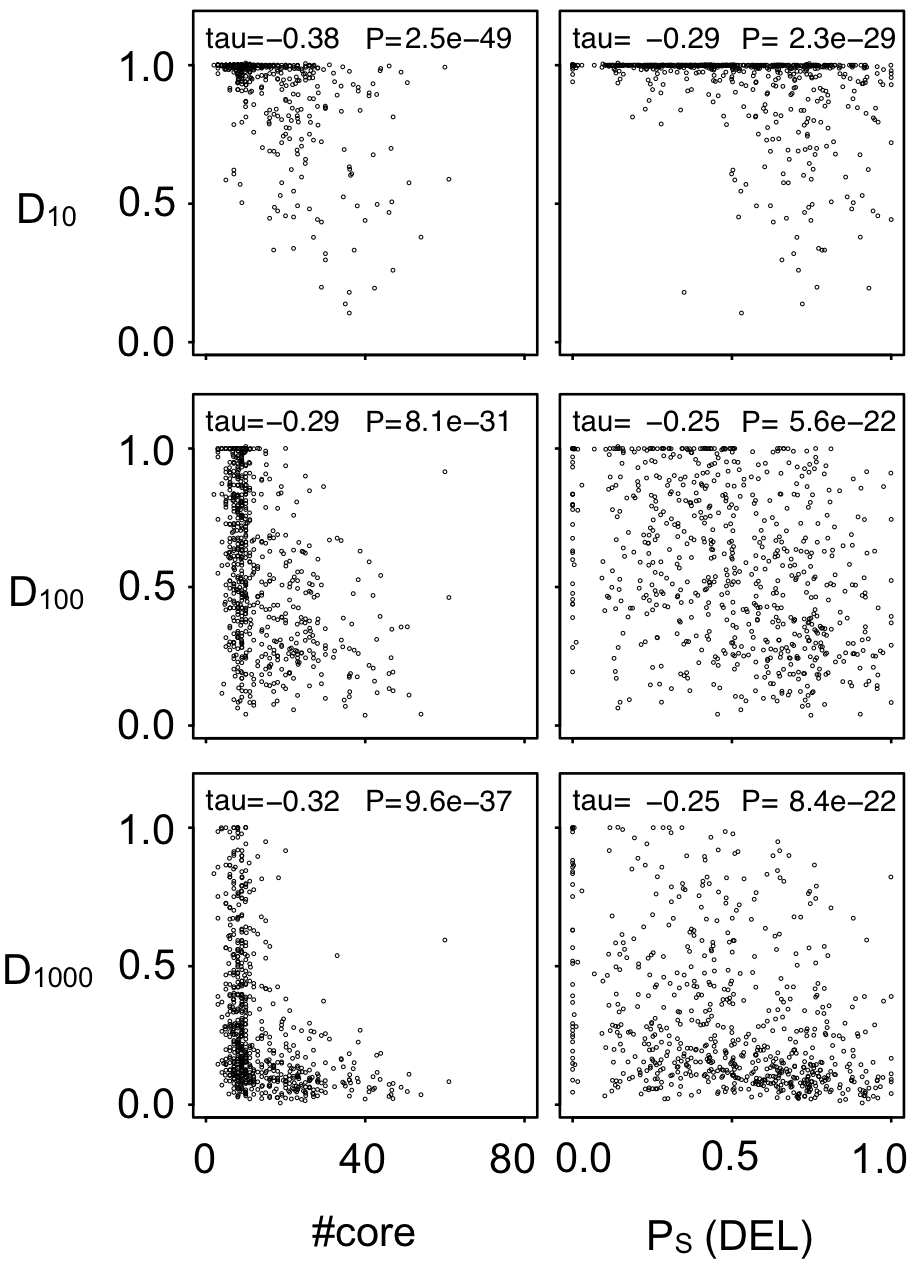

Supplement: Figure S1 — Relationship between the rate of phenotypic adaptation and the properties of GRNs. After 50,000 generations in the experimental evolution, a new optimum was placed at a constant distance away (d = 1) from the mean phenotype of the population. The population was then allowed to evolve for 1000 generations (denoted as the benchmark evolution). Points represent the results of each population in the random-walk optimum shift. Horizontal axes indicate the number of core genes and PS of a population at the end of experimental evolution. Di indicates the Euclidean distance between the optimum and the mean phenotype of a population at generation i during the benchmark evolution. Kendall's correlation test was used for statistical analysis of the correlation. (0.31 MB TIF) [file pcbi.1000873.s001.tif]

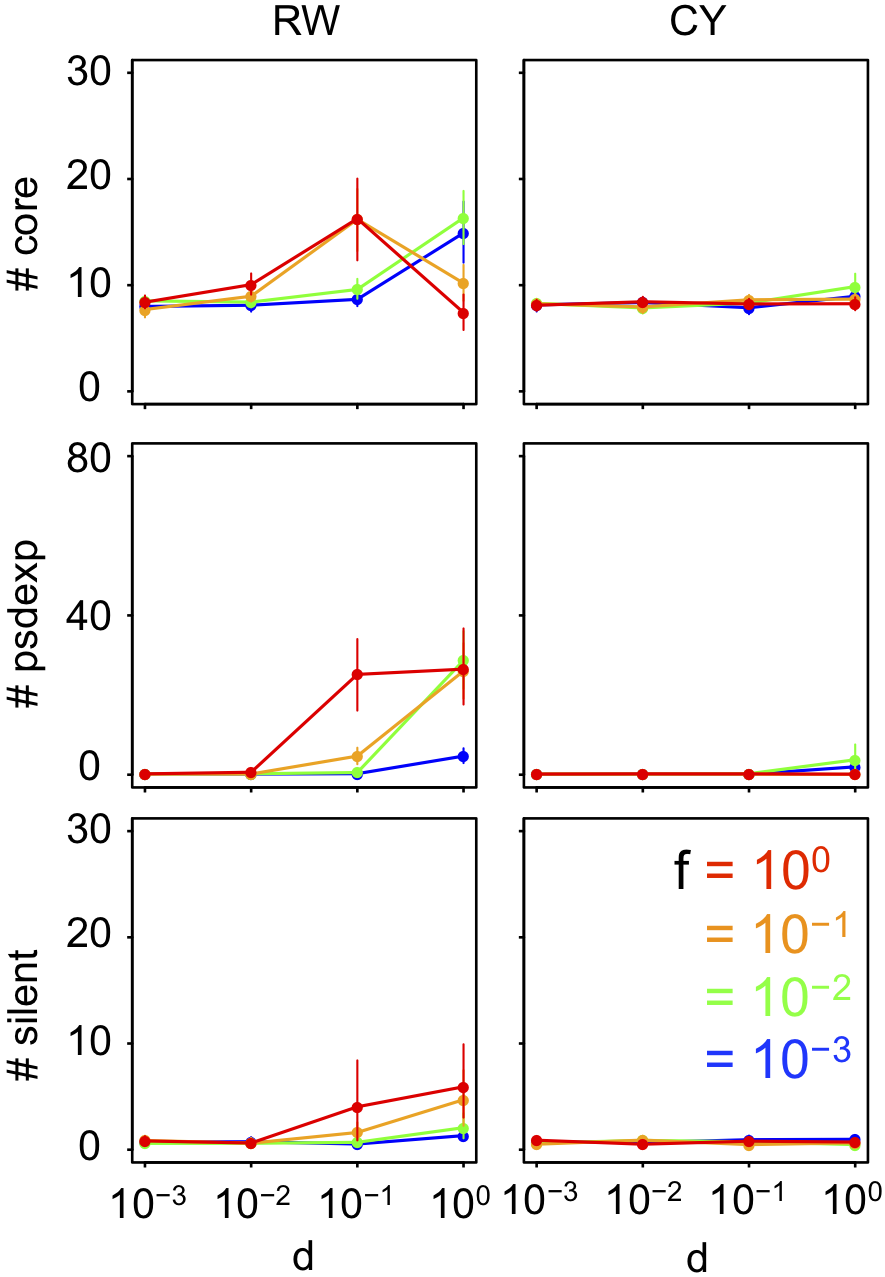

Supplement: Figure S2 — Number of regulatory genes in GRN that evolved under fixed per-individual mutation rate. GRNs were allowed to evolve with a fixed per-individual mutation rate regardless of the number of genes in GRNs. μBTL = μCIS = μTRA = μDEL = μDUP = 10−5 per-individual per generation. Each point connected by solid lines represents the mean number of each type of genes in evolved GRNs under each selective condition. Vertical bars attached to the point represent 95% confidence intervals. d and f represent the amplitude and frequency of the optimum shift, respectively. (0.16 MB TIF) [file pcbi.1000873.s002.tif]

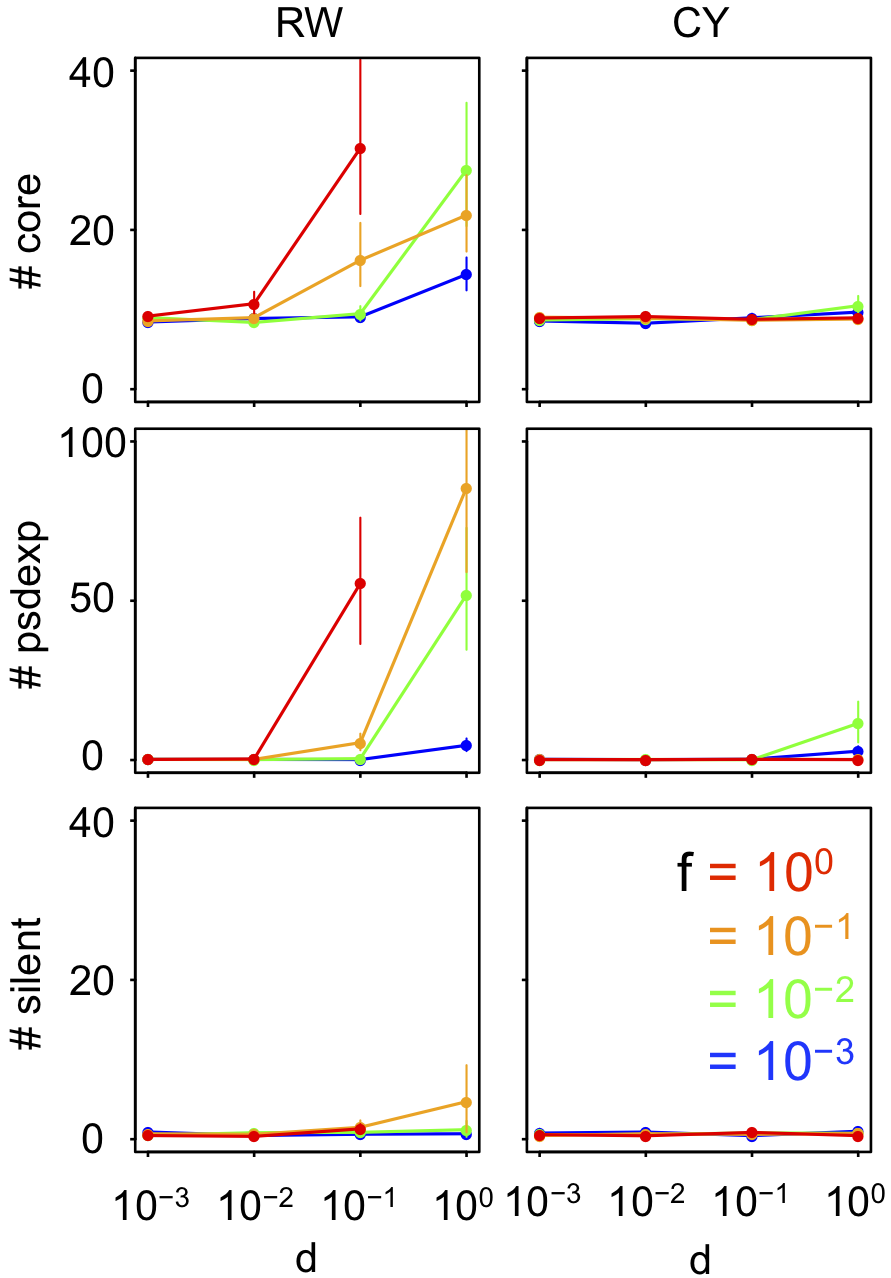

Supplement: Figure S3 — Number of regulatory genes in GRN that evolved under constant PS. GRNs were allowed to evolve with a constant PS regardless of the number of genes in GRNs (PS = 1, PL = PN = 0). μBTL = μCIS = μTRA = μDEL = μDUP = 10−5 per-individual per generation. Each point connected by solid lines represents the mean number of each type of genes in evolved GRNs under each selective condition. Vertical bars attached to the point represent 95% confidence intervals. d and f represent the amplitude and frequency of the optimum shift, respectively. (0.16 MB TIF) [file pcbi.1000873.s003.tif]

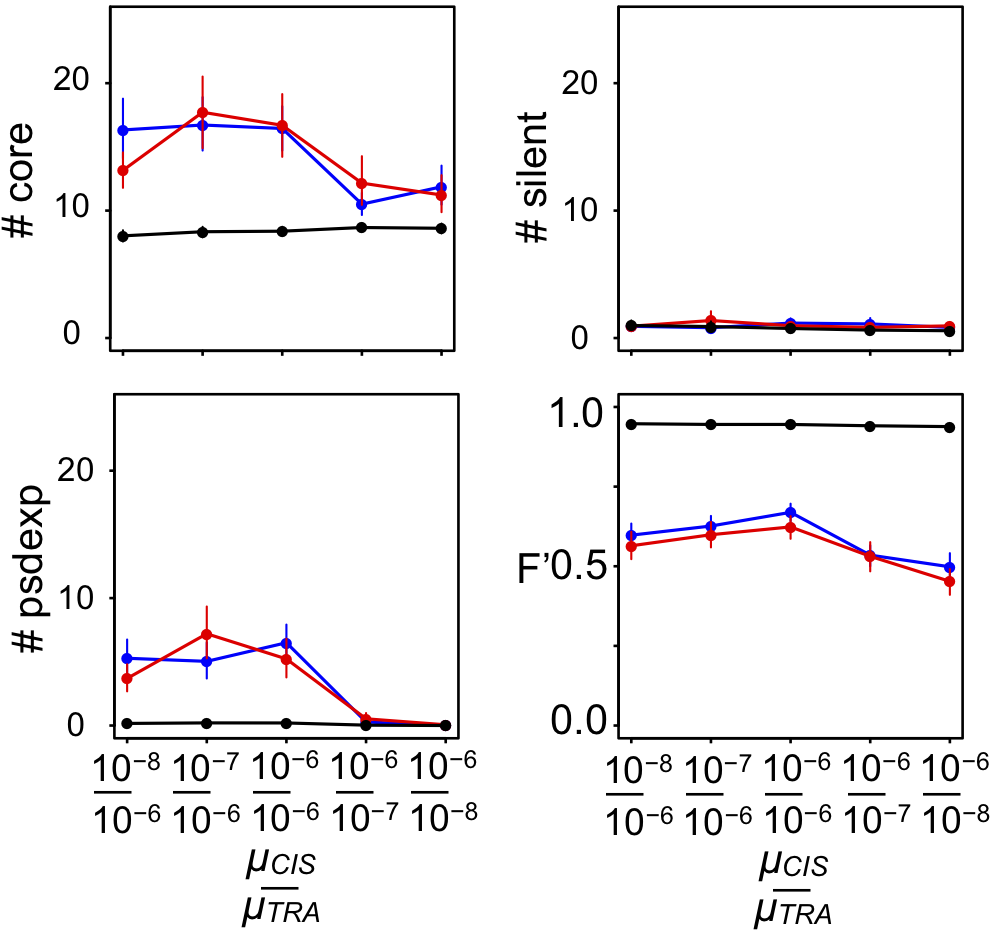

Supplement: Figure S4 — Number of regulatory genes in GRN that evolved under various rates of cis- and trans-regulatory mutations (μCIS, μTRA). The values of both μCIS and μTRA are varied from 10−8 to 10−6 (μCIS = μTRA = 10−6, standard parameter value). Points connected by solid lines represent the mean number of core genes (#core), pseudo-expression genes (#psdexp), and silent genes (#silent) in GRNs that evolved for 50,000 generations under each simulation condition. Vertical bars indicate 95% confidence intervals. Different colors indicate different conditions of phenotypic selection; d = 10−1, f = 10−1 (red); d = 100, f = 10−3 (blue); d = 10−3, f = 10−3 (black) under random-walk optimum shift. (0.13 MB TIF) [file pcbi.1000873.s004.tif]

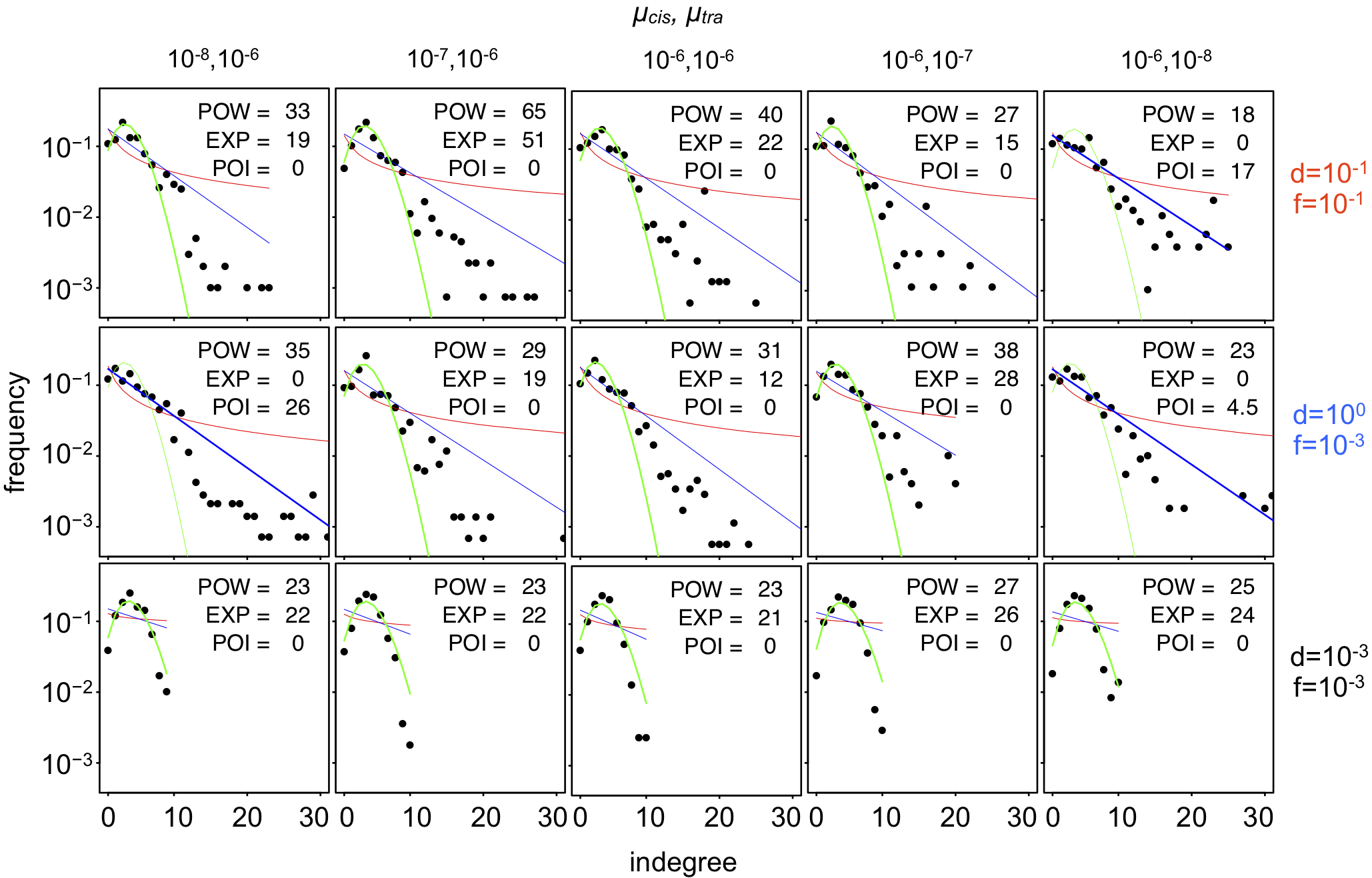

Supplement: Figure S5 — Indegree distribution of assembled GRNs that evolved under various rates of cis- and trans-regulatory mutations (μCIS, μTRA). The values of both μCIS and μTRA are varied from 10−8 to 10−6, standard parameter value). Horizontal and vertical axes in each panel show the indegree (the number of regulatory interactions that arrived at a gene) and the frequency, respectively. Note that the vertical axes are shown logarithmically to demonstrate the exponential character of the distribution. Different rows and columns show the indegree distributions of GRNs under different conditions of phenotypic selection and different values of (μCIS, μTRA), respectively. Lines in each panel indicate the regression of the plot to the Power law distribution (red), the exponential distribution (blue) and the Poisson distribution (green). Regression was estimated by a nonlinear least-square method. To judge the goodness of the regression, Akaike's information criterion (AIC) was used, and the regression that showed the smallest value of AIC was drawn as a thick line. POW, EXP and POI in each panel indicate the differences between AIC value of the best regression model and those of power-law (scale-free), exponential and poisson distributions, respectively. (0.52 MB TIF) [file pcbi.1000873.s005.tif]

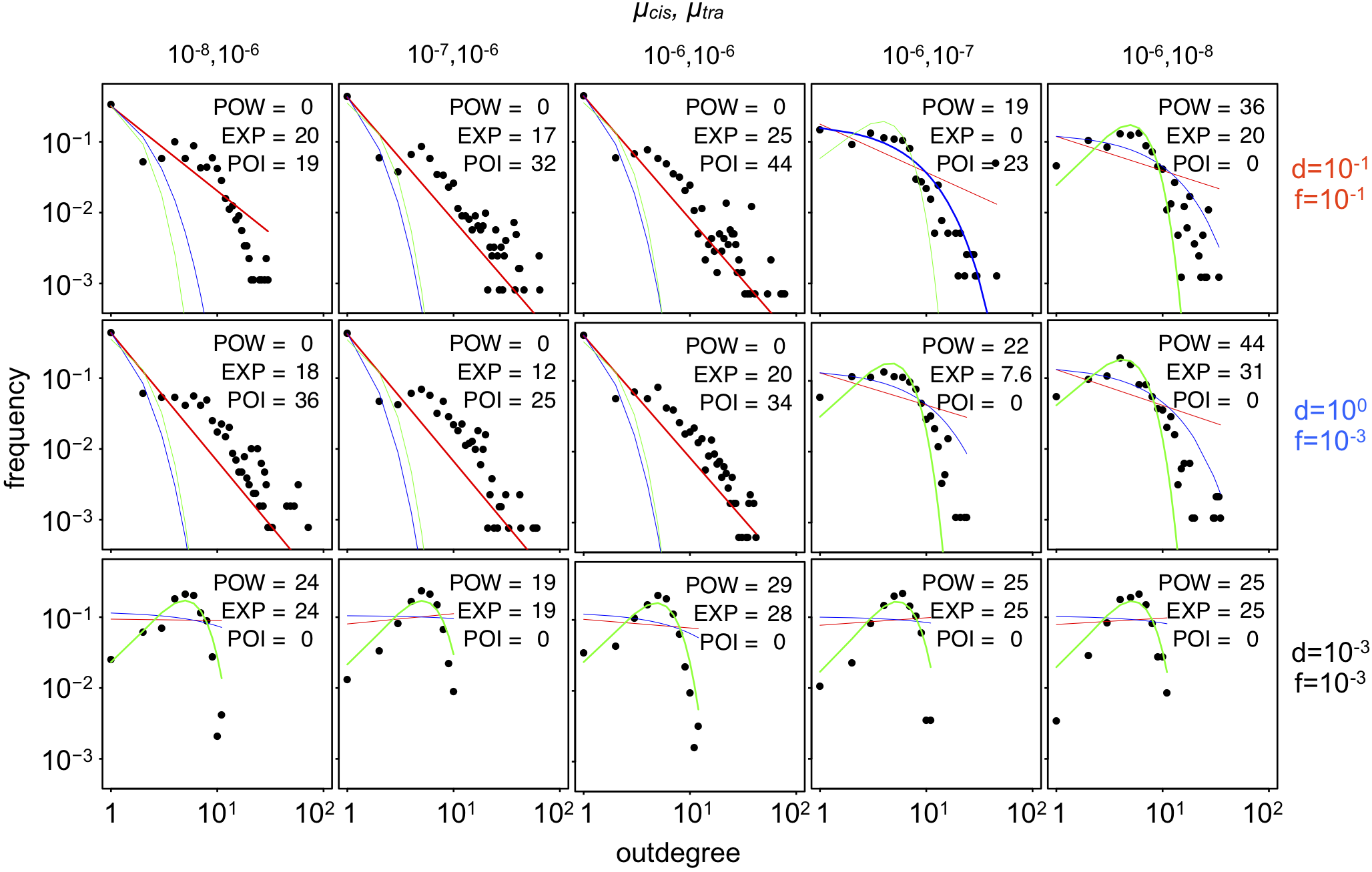

Supplement: Figure S6 — Outdegree distribution of assembled GRNs that evolved under various rates of cis- and trans-regulatory mutations (μCIS, μTRA). The values of both μCIS and μTRA are varied from 10−8 to 10−6 (μCIS = μTRA = 10−6, the standard parameter value). Horizontal and vertical axes in each panel show the outdegree (the number of regulatory interactions that depart from a gene) and the frequency, respectively. Note that the both horizontal and vertical axes are shown logarithmically to demonstrate the scale-free character of the distribution. Different rows and columns show the outdegree distributions of GRNs under different conditions of phenotypic selection and different values of (μCIS, μTRA), respectively. Lines in each panel indicate the regression of the plot to the Power law distribution (red), exponential distribution (blue) and Poisson distribution (green). Regression was estimated by a nonlinear least-square method. To judge the goodness of the regression, Akaike's information criterion (AIC) was used, and the regression that showed the smallest value of AIC was drawn as a thick line. POW, EXP and POI in each panel indicate the differences between AIC value of the best regression model and those of power-law (scale-free), exponential and poisson distributions, respectively. (0.55 MB TIF) [file pcbi.1000873.s006.tif]

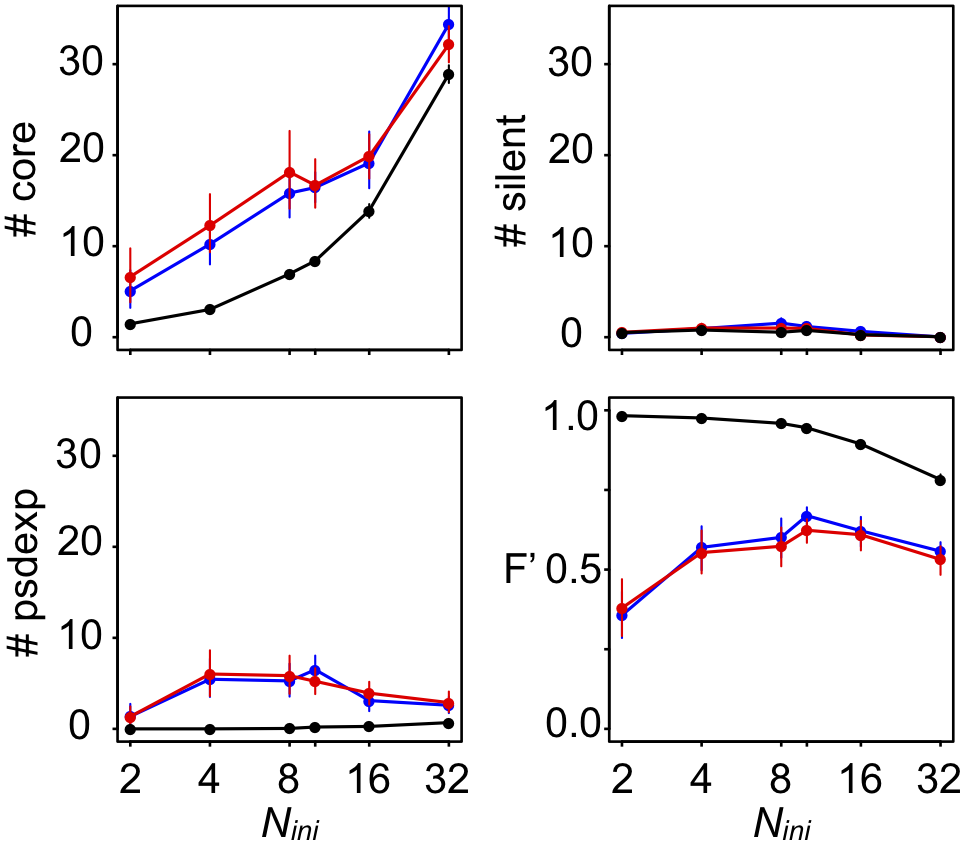

Supplement: Figure S7 — Number of regulatory genes in GRN that evolved under the various initial numbers of regulatory genes (Ninit). Ninit = 10, standard parameter value. Points connected by solid lines represent the mean number of core genes (#core), pseudo-expression genes (#psdexp), and silent genes (#silent) in GRNs that evolved for 50,000 generations under each simulation condition, respectively. Vertical bars indicate 95% confidence intervals. Different colors indicate the different conditions of phenotypic selection; d = 10−1, f = 10−1 (red); d = 100, f = 10−3 (blue); d = 10−3, f = 10−3 (black) under random-walk optimum shift. (0.13 MB TIF) [file pcbi.1000873.s007.tif]

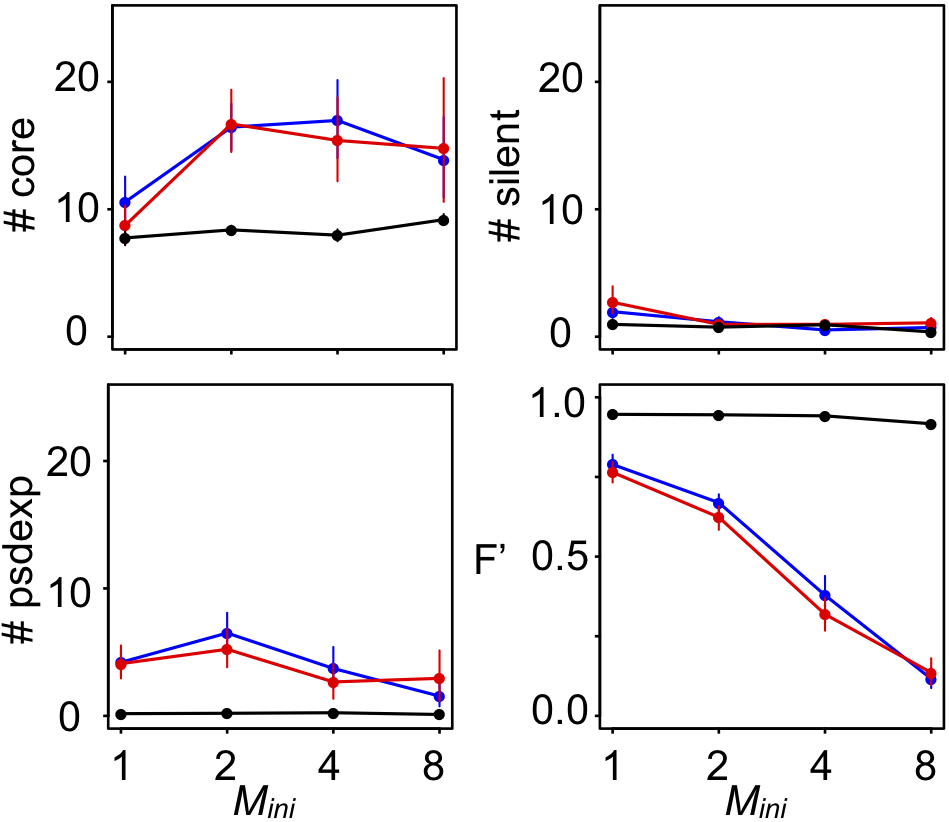

Supplement: Figure S8 — Number of regulatory genes in GRN that evolved under various initial numbers of phenotypic genes (Minit). Minit = 2, standard parameter value. Points connected by solid lines represent the mean number of core genes (#core), pseudo-expression genes (#psdexp), and silent genes (#silent) in GRNs that evolved for 50,000 generations under each simulation condition, respectively. Vertical bars indicate 95% confidence intervals. Different colors indicate the different conditions of phenotypic selection; d = 10−1, f = 10−1 (red); d = 100, f = 10−3 (blue); d = 10−3, f = 10−3 (black) under random-walk optimum shift. (0.11 MB TIF) [file pcbi.1000873.s008.tif]

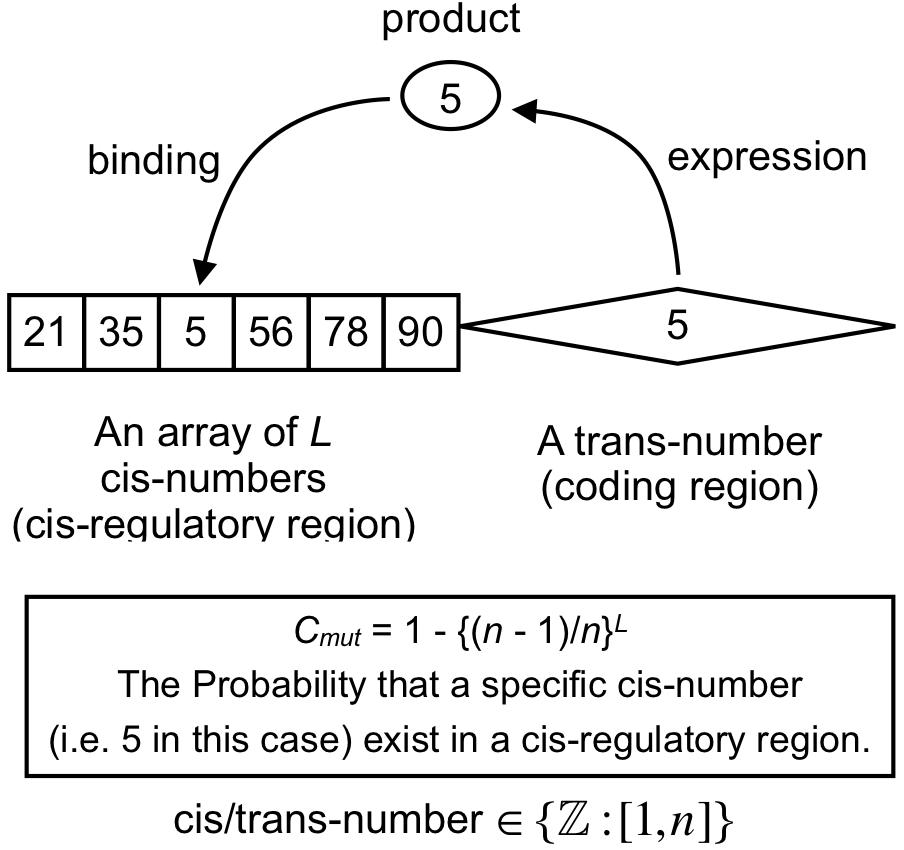

Supplement: Figure S9 — Relationship between gene structures and Cmut. (0.14 MB TIF) [file pcbi.1000873.s009.tif]

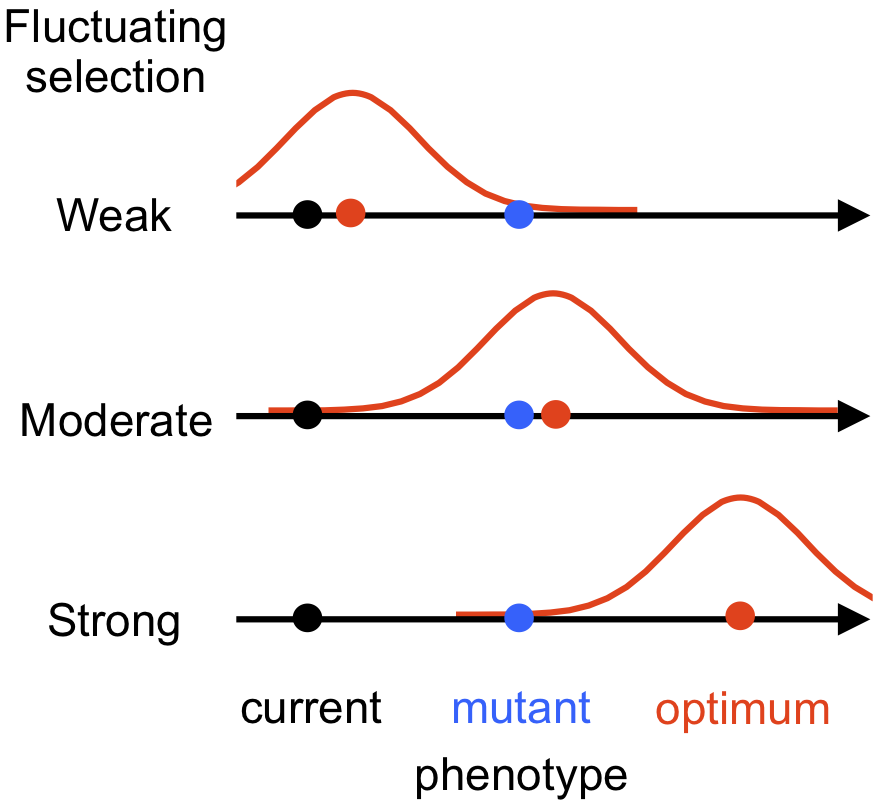

Supplement: Figure S10 — Illustration of the relationship between the intensity of optimum fluctuation and the fitness effects of a certain mutation. (0.09 MB TIF) [file pcbi.1000873.s010.tif]
